# Association between wine consumption and cancer: a systematic review and meta-analysis

**DOI:** 10.3389/fnut.2023.1197745

**Published:** 2023-09-04

**Authors:** Maribel Lucerón-Lucas-Torres, Iván Cavero-Redondo, Vicente Martínez-Vizcaíno, Bruno Bizzozero-Peroni, Carlos Pascual-Morena, Celia Álvarez-Bueno

**Affiliations:** ^1^Health and Social Research Center, Universidad de Castilla-La Mancha, Cuenca, Spain; ^2^Facultad de Ciencias de la Salud, Universidad Autónoma de Chile, Talca, Chile; ^3^Higher Institute of Physical Education, Universidad de la República, Rivera, Uruguay; ^4^Universidad Politécnica y Artística del Paraguay, Asunción, Paraguay

**Keywords:** cancer, wine, adult people, alcohol consumption, systematic review and meta-analysis

## Abstract

**Background:**

Alcohol consumption is related to the risk of developing different types of cancer. However, unlike other alcoholic beverages, moderate wine drinking has demonstrated a protective effect on the risk of developing several types of cancer.

**Objective:**

To analyze the association between wine consumption and the risk of developing cancer.

**Methods:**

We searched the MEDLINE (through PubMed), Scopus, Cochrane, and Web of Science databases to conduct this systematic review and meta-analysis. Pooled relative risks (RRs) were calculated using the DerSimonian and Laird methods. I2 was used to evaluate inconsistency, the τ2 test was used to assess heterogeneity, and The Newcastle-Ottawa Quality Assessment Scale were applied to evaluate the risk of bias. This study was previously registered in PROSPERO, with the registration number CRD42022315864.

**Results:**

Seventy-three studies were included in the systematic review, and 26 were included in the meta-analysis. The pooled RR for the effect of wine consumption on the risk of gynecological cancers was 1.03 (95% CI: 0.99, 1.08), that for colorectal cancer was 0.92 (95% CI: 0.82, 1.03), and that for renal cancer was 0.92 (95% CI: 0.81, 1.04). In general, the heterogeneity was substantial.

**Conclusion:**

The study findings reveal no association between wine consumption and the risk of developing any type of cancer. Moreover, wine drinking demonstrated a protective trend regarding the risk of developing pancreatic, skin, lung, and brain cancer as well as cancer in general.

**Systematic review registration:**

https://www.crd.york.ac.uk/prospero/display_record.php?ID=CRD42022315864, identifier CRD42022315864 (PROSPERO).

## Introduction

Cancer is associated with some of the highest mortality and morbidity rates worldwide, surpassed only by cardiovascular diseases (1); an estimated 23.6 million cancer cases and 10 million cancer-related deaths occurred in 2019. Moreover, the incidence of cancer is expected to increase due to the decline in cancer screening and early diagnosis brought about by COVID-19 (2, 3). The main cancers causing disability-adjusted life years in both sexes affect the respiratory tract, such as the trachea, bronchus, and lung; the colon, rectum, stomach, breast, and liver are also common cancer sites, and breast cancer is the leading cause of cancer-related death in women (2, 4).

The main risk factors affecting the overall burden of cancer are smoking, alcohol consumption, and high body mass index (4), but non-modifiable risk factors such as genetics and age should not be overlooked considering that we will never be able to reduce their burden on cancer even if we try to address modifiable risk factors (5). Tobacco consumption appears to be the major risk factor for lung cancer (6), and studies have shown a concomitant effect of tobacco and alcohol consumption on some cancers (7). Additionally, alcohol consumption is associated with an increased risk for upper aerodigestive tract, colon, rectum, liver, head, neck, and breast cancer in women (8, 9), responsible for approximately 6 million new cancer cases and 3 million deaths in 2020 (10). However, the incidence of kidney cancers appears inversely associated with moderate alcohol consumption (11) (20 g/day (2 SBU) for men and 10 g/day (1 SBU) for women (12)), as reported by the World Health Organization.

Current knowledge regarding alcohol consumption and cancer remains controversial, specifically in relation to wine consumption. Some previous meta-analyses revealed no association between wine consumption and cancer but an increased risk of cancer with the consumption of other alcoholic beverages (13) and suggested that beer is associated with the highest risk for colorectal cancer (14). Regarding gynecological cancers, alcohol consumption may increase the incidence of both breast and ovarian cancer, although the role of wine consumption has not been clearly determined (15). Other risk factors for these cancers should be considered; age at menarche and BRCA gene carrier status for ovarian and breast cancer (16), and tubal ligation history and menopause age for ovarian cancer (17). Furthermore, unlike other alcoholic beverages, moderate wine consumption has shown a protective effect on the likelihood of developing different types of cancer, such as rectal (18, 19) and colorectal (19, 20) cancer. Other studies have found that wine consumption is not a risk factor for esophageal (21) or lung (22) cancer.

Due to the inconsistencies in the data regarding the relationship between wine consumption and cancer development and the increasing interest in the effects of wine on health, this systematic review and meta-analysis was conducted to summarize the existing evidence on various cancers and analyze the effect of wine consumption independently from other alcoholic beverages. This is the first systematic review and meta-analysis on this topic, and the aim was to analyze the association between wine consumption and general, upper digestive tract, colorectal, renal, pancreatic, skin, lung, brain, and gynecological cancer.

## Methodology

### Search strategy and selection of studies

This systematic review and meta-analysis was conducted following the Cochrane Collaboration Handbook (23) and in accordance with the guidelines of the Meta-analysis of Observational Studies in Epidemiology statement (MOOSE) (24). This study was previously registered in PROSPERO, with the registration number CRD42022315864.

A systematic search of the MEDLINE (via PubMed), Scopus, Cochrane, and Web of Science databases was conducted from their inception until 12 December 2022. The search strategy followed the PICO structure (population, intervention/exposure, comparison, outcome, and study design), using Boolean operators between the following terms: “adults,” “young adults,” “adult populations,” “adult subjects,” “older,” “elderly,” “elderly people,” “older people,” “alcohol,” “wine,” “alcohol consumption” “wine consumption,” “neoplasm,” “cancer,” “tumor,” “cancer risk,” “carcinogen*,” “mortalit*,” “cohort,” “cases and controls,” “longitudinal studies,” and “prospective studies” (Supplementary Table S1). In addition, references from previous systematic reviews and meta-analyses were reviewed.

### Eligibility

The articles included in this systematic review and meta-analysis were longitudinal studies measuring the association between wine consumption and different types of cancer, including general, upper digestive tract, colorectal, renal, pancreatic, skin, lung, brain, and gynecological cancer. The inclusion criteria were as follows: (i) participants: general population; (ii) exposure: wine consumption as reported by the original studies; (iii) outcomes: different types of cancer; and (iv) study design: longitudinal studies (cohort and case–control studies). Conversely, studies were excluded when (i) they were review studies, ecological studies, editorials, or case reports; (ii) they were not written in English or Spanish; or (iii) they did not report wine consumption separately from other alcoholic beverages. No publication date restriction was applied.

### Data extraction and quality assessment

The following information was extracted from the included studies and synthesized in an *ad hoc* table (Table 1): (1) reference: first author and year of publication; (2) country; (3) study design; (4) participant characteristics: sample size, percentage of women, age, and type of population; (5) exposure follow-up in years (if reported); and (6) outcome: type of cancer. In addition, covariables used in the analyses of the included studies were summarized in an additional table since each study included different confounding variables.

**Table 1:** Main characteristics of the included studies.

References	Country	Design of study	Characteristics of the participants			Follow up (years)	Outcome
			N, women (%)	Age*	Target population		Type of cancer
Breast cancer							
Harvey et al., 1986	USA, America	Case–control	Cases: 1524 (100) Controls: 1896 (100)	30–50	General population	5	Breast cancer
Howe al., 1991	Multicenter (Australia, Canada, Greece, Argentina and Italy)	Case–control	Cases: 1575 Controls: 1974	<30– >82	General population	3	Breast cancer
Sneyd et al., 1991	New Zealand	Case–control	Cases: 891 Controls:1864	25–54	General population	4	Breast cancer
Friedenreich et al., 1993	Canada, America	Case–control	N: 1701 Cases: 519 Controls: 1182	NR	General population	5	Breast cancer
Longnecker et al., 1995	USA, America	Case–control	Cases: 6888 Controls: 9424	58.7	General population	3	Breast cancer
Swanson et al., 1996	USA, America	Case–control	Cases: 1668 (100) Controls:1501 (100)	Cases: 40 (NR) Controls: 39 (NR)	General population	2	Breast cancer
Zhang et al., 1999	USA, America	Cohort	Framingham Study: 2873 Framingham Offspring Study: 5124	-Framingham Study: 68.4 (NR) -Framingham Offspring Study: 54.2 (NR)	General population	-Framingham Study: 34.3 -Framingham Offspring Study: 19.3	Breast cancer
Rohan et al., 2000	Canada, America	Cohort	56,837 (100)	40–59	NBSS	5	Breast cancer
Horn-Ross et al., 2002	USA, America	Cohort	111,526	21–103	California Teachers Study cohort	2	Breast cancer
Tjønneland et al., 2007	Europe	Cohort	368,010 (100)	35–70	EPIC cohort	6.4	Breast cancer
Li et al., 2009	USA, America	Cohort	70,033	40.6 (NR)	Multi-ethnic cohort. Members of a comprehensive pre-paid health care programme in the San Francisco Bay Area.	16	Breast cancer
Prostate cancer							
Tavani et al., 1994	Italy, Europe	Case–control	Cases: 281 Controls: 599	Cases: 67 (NR) Controls: 63 (NR)	General population	7	Prostate cancer
De Stefani et al., 1995	Uruguay, America	Case–control	Cases: 156 Controls: 302	Cases: 40–89 Controls: 40–89	General population	6	Prostate cancer
Hayes et al., 1996	USA, America	Case–control	Cases: 1292 Controls: 1767	40–79	General population	4	Prostate cancer
Andersson et al., 1996	Sweden, Europe	Case–control	Cases:256 Controls:252	Cases: 70.0 (6.1) Controls: 69.8 (6.2)	General population	3	Prostate cancer
Jain et al., 1998	Canada, America	Case–control	Cases: 617 Controls: 637	Cases: 69.8 (7.3) Controls: 69.9 (7.3)	General population	4	Prostate cancer
Schuurman et al., 1999	Netherlands, Europe	Cohort	58,279	55–69	NLCS	6.3	Prostate cancer
Breslow et al., 1999	USA, America	Cohort	3,775	25–74	NHEFS Cohort	9	Prostate cancer
Putnam et al., 2000	USA, America	Cohort	1,577	68.1 (NR)	Cohort of Iowa men	3	Prostate cancer
Barba et al., 2004	USA, America	Case–control	Cases: 88 Controls: 272	Cases: 69.3 (8.4) Controls: 70.0 (6.3)	The PROMEN STUDY	4	Prostate cancer
Crispo et al., 2004	Italy, Europe	Case–Control	2,663	Prostatic carcinoma: 66 (NR) Benign prostatic hyperplasia: 65 (NR)	General population	12	-Prostate cancer -Benign prostatic hyperplasia
Platz et al., 2004	USA, America	Cohort	47,843	54.7 (NR)	Health Professionals Follow-up Study	12	Prostate cancer
Chang et al., 2005	Sweden, Europe	Case–control	Cases: 1130 Controls:1499	Cases: 66.4 (7.3) Controls: 67.3 (7.6)	General population	2	Prostate cancer
Schoonen et al., 2005	USA, America	Case–control	Cases: 753 Controls: 703	40–64	Caucasian and African-American	4	Prostate cancer
Sutcliffe et al., 2007	USA, America	Cohort	45,433	53.8 (NR)	Health Professionals Follow-up Study	4	Prostate cancer
Renal cell cancer							
Pelucchi et al., 2002	Italy, Europe	Case–control	Cases: 348 (32.18) Controls: 1048 (28.15)	Cases: 60 (NR) Controls: 60 (NR)	General population	8	Renal cell cancer
Rashidkhani et al., 2005	Sweden, Europe	Cohort	59,237 (100)	40–76	Swedish Mammography Cohort	3	Renal cell cancer
Greving et al., 2007	Sweden, Europe	Case–control	Cases: 855 Control: 1204	Cases: 64.3 (NR) Controls: 64.4 (NR)	General population	3	Renal cell cancer
Lew et al., 2011	USA, America	Cohort	492.187 (40.4)	62.1 (NR)	NIH-AARP Diet and Health study	9	Renal cell cancer
Pancreatic cancer							
Tavani et al., 1997	Italy, Europe	Case–control	Cases: 361 Control: 997	Cases: 60 (NR) Controls: 59 (NR)	General population	10	Pancreatic cancer
Michaud et al., 2001	USA, America	Cohort	HPFS: 10582 NHS:30083	HPFS: 54.5 (NR) NHS: 47.5 (NR)	Health Professionals Follow-Up Study and Nurses’ Health Study	4	Pancreatic cancer
Heinen et al., 2009	Netherlands, Europe	Cohort	120,852 (50.52)	62.1 (4.1)	Netherlands Cohort Study	13.3	Pancreatic cancer
Jiao et al., 2009	USA, America	Cohort	470,681 (40.5)	62 (NR)	The NIH-AARP Diet and Health Study	7.3	Pancreatic cancer
Gapstur et al., 2011	USA, America	Cohort	1,029,467 (56.02)	33–111	The Cancer Prevention Study II	24	Pancreatic cancer
Ovarian cancer							
Gwinn et al., 1986	USA, America	Case–control	Cases: 433 Controls: 2915	20–54	General population	2	Ovarian cancer
La Vecchia et al., 1992	Italy, Europe	Case–control	Cases: 801 Controls: 2114	54 (NR)	General population	7	Ovarian cancer
Tavani et al., 2001	Italy, Europe	Case–control	Cases: 1031 Controls: 2411	Cases: 18–79 Controls: 17–79	General population	7	Ovarian cancer
Webb et al., 2003	Australia, Oceania	Case–control	Cases: 696 (100) Controls: 786 (100)	18–79	General population	3	Ovarian cancer
Goodman et al., 2003	USA, America	Case–control	Cases: 558 Controls: 607	54.8 (NR)	General population	6	Ovarian cancer (general): -Mucinous -Nonmucinous Invasive ovarian cancer: -Serous -Endometrioid -Mucinous
Modugno et al., 2003	USA, America	Case–control	Cases: 761 -Nonmucinous cases: 649 -Mucinous cases: 112 Controls: 1352	20–69	Study of Health and Reproduction Project	5	Ovarian cancer: -Nonmucinous -Mucinous
Schouten et al., 2004	Netherlands, Europe	Cohort	62,573	61.3 (4.2)	NLCS	9.3	Ovarian cancer
Peterson et al., 2006	USA, America	Case–control	Cases: 762 Controls: 6271	Cases: 57.6 (NR) Controls: 59.8 (NR)	General population	3	Ovarian cancer
Chang et al., 2007	USA, America	Cohort	90,371 (100)	50 (NR)	The CTS cohort	8.1	Ovarian cancer
Cook et al., 2016	Canada, America	Case–control	Cases: 1144 (100) Controls: 2513 (100)	Cases: 59.6 (9.8) Controls: 57.1 (9.1)	The OVAL-BC Study	11	Ovarian cancer
Aerodigestive tract cancers							
Franceschi et al., 1990	Italy, Europe	Case–control	Cases: -Oral cavity: 157 -Pharyns: 134 -Larynx: 162 -Esophagus: 288 Controls: 1272	<49– > 70	General population	3	-Oral cavity -Pharynx -Larynx -Esophagus
Barra et al., 1990	Italy, Europe	Case–control	Oral cavity: 305 esophageal: 288 Controls: 1621	Oral cavity: 58 (NR) esophageal: 60 (NR) Controls: 57 (NR)	General population	5	-Oral cavity -esophagus
Barra et al., 1991	Italy, Europe	Case–control	Cases: 272 Control: 445	Cases: 60 (NR) Controls: 57 (NR)	General population	5	-Oral cavity-pharynx
De Stefani et al., 1998	Uruguay, America	Case–control	Cases: 471 Controls: 471	Cases 40–89 Controls 40–89	General population	4	-Oral cavity-pharynx
Grønbæk et al., 1998	Denmark, Europe	Cohort	28,180 (46.4)	20–98	The Copenhagen Centre for Prospective Population Studies	13.5	Upper digestive tract cancer
Huang et al., 2003	Puerto Rico, America	Case–control	Cases: 286 Controls: 417	21–79	General population	3	Oral cancer study
Aerodigestive tract cancers							
Barstad et al., 2005	Denmark, Europe	Cohort	-Copenhagen City Heart Study: 15.754 (53.8) -Copenhagen Male study: 3230 (0) -Copenhagen Country Centre of Preventive Medicine 1897 cohort: 243 (58.4) -Copenhagen Country Centre of Preventive Medicine 1914 cohort: 933 (49.6) -Copenhagen Country Centre of Preventive Medicine 1936 cohort: 1107 (41.8) -MONICA I: 3774 (48.8) -MONICA II: 1413 (49.7) -MONICA III:2009 (50.5)	-Copenhagen City Heart Study: 53 (NR) -Copenhagen Male study: 63 (NR) -Copenhagen Country Centre of Preventive Medicine 1897 cohort: 80 (NR) -Copenhagen Country Centre of Preventive Medicine 1914 cohort: 70 (NR) -Copenhagen Country Centre of Preventive Medicine 1936 cohort: 40 (NR) -MONICA I: 46 (NR) -MONICA II: 45 (NR) -MONICA III: 50 (NR)	-Copenhagen City Heart Study -Copenhagen Male study -Copenhagen Country Centre of Preventive Medicine 1897 cohort -Copenhagen Country Centre of Preventive Medicine 1914 cohort -Copenhagen Country Centre of Preventive Medicine 1936 cohort -MONICA I -MONICA II -MONICA III	-Copenhagen City Heart Study: 16.0 -Copenhagen Male study: 9.8 -Copenhagen Country Centre of Preventive Medicine 1897 cohort: 7.3 -Copenhagen Country Centre of Preventive Medicine 1914 cohort: 9.0 -Copenhagen Country Centre of Preventive Medicine 1936 cohort: 18.7 -MONICA I: 13.0 -MONICA II: 9.8 -MONICA III: 5.3	Gastric cancer risk
Pandeya et al., 2009	Australia, Oceania	Case–control	-EAC: 365 (9.7) -EGJAG: 426 (13.2) -ESCC: 303 (43.1) -Controls: 1580 (34.2)	-EAC: 63.6 (0.5) -EGJAG: 63.3 (0.5) -ESCC: 64.7 (0.5) -Controls: 60.5 (0.3)	General population	3	-EAC -EGJAG -ESCC
Skin carcinoma							
Fung et al., 2002	USA, America	Cohort	NHS: 3060 HPFS: 3028	NHS: 30–55 HPFS: 40–75	Health Professionals Follow-Up Study and Nurses’ Health Study	NHS: 8 HPFS: 10	Skin carcinoma
Ansems et al., 2008	Australia, Oceania	Cohort	1,360 (57.6)	49.7 (NR)	The Nambour Skin Cancer Study	11	-BCC -SCC
Lung cancer							
Benedetti et al., 2006	Canadá, America	Case–control	Study I Cases: 699 Controls: 507 Study II Men Cases: 640 Controls: 861 Study II Women Cases: 454 Controls: 607	Study I Cases: 59.0 (7.8) Controls: 59.7 (7.8) Study II Men Cases: 63.8 (8.0) Controls: 63.2 (7.7) Study II Women Cases: 60.9 (9.4) Controls: 60.7 (9.1)	Study I: Men Study II: Men and women.	10	Lung cancer
Colorectal cancer							
Potter et al., 1986	Australia, Oceania	Case–control	COLON CANCER Cases: 220 (45) Controls: 438 (45) RECTUM CANCER Cases: 199 (37.7) Controls: 396 (37.4)	30–74	General population	3	-Colon cancer -Rectum cancer
Longnecker et al., 1990	USA, America	Case–control	RIGHT COLON Cases: 251 Controls: 367 RECTUM Cases: 393 Controls: 625	<60– > 80	Males	3	-Right colon cancer -Rectum cancer
Freudenheim et al., 1990	USA, America	Case–control	Cases: 422 (34.4) Controls: 844	>40	General population	8	-Rectal cancer
Meyer et al., 1993	USA, America	Case–control	Cases: 424 Controls: 414	Cases: 54.9 (NR) Controls: 54.4 (NR)	General population	4	-Colon cancer
Newcomb et al., 1993	USA, America	Case–control	Cases: 779 Controls: 2315	Cases: 65.5 (8.8) Controls: 58.6 (10.9)	Females	1	-Colon cancer -Rectal cancer
Gapstur et al., 1994	USA, America	Cohort	41,837	55–69	General population	5	-Proximal colon -Distal colon -Rectal
Goldbohm et al., 1994	Netherlands, Europe	Cohort	120,852 (50.52)	55–69	General population	3.3	-Colon -Rectal
Colorectal cancer							
Sharpe et al., 2002	Canada, America	Case–control	Cases: 585 -Proximal colon: 176 -Distal colon: 179 -Rectum: 230 Controls: 500	35–70	General population	6	-Proximal colon -Distal colon -Rectum
Pedersen et al., 2003	Denmark, Europe	Cohort	29,132 (46.82)	23–95	The Copenhagen Centre for Prospective Population Studies is based on three comprehensive Danish programmes of prospective population studies: the Copenhagen City Heart Study, the Copenhagen County Centre of Preventive Medicine (formerly, the Glostrup Population Studies) which includes six cohorts, and the Copenhagen Male Study.	14.7	-Colon cancer -Rectal cancer
Bongaerts et al., 2008	Netherlands, Europe	Cohort	120,852 (50.52)	55–69	General population	13.3	-Overall colorectum -Colon -Proximal colon -Distal colon -Rectosigmoid -Rectum
Crockett et al., 2011	USA, America	Case–control	Cases: 1033 (43.5) Controls: 1011 (41.0)	Cases: 40–79 Controls: 40–79	NCCCS-II	5	-Rectum cancer -Rectosigmoid cancer -Sigmoid cancer
Cancer in general							
Gong et al., 2009	USA, America	Cohort	10,920 (0)	<60, 60–69, and ≥ 70	General population	7	Cancer in general
Smyth et al., 2015	Canada, America	Cohort	155,875	35–70	PURE study	4.3	Cancer in general
Schutte et al., 2021	United Kingdom, Europe	Cohort	446,439 (53.8)	56.4 (8.1)	General population	4	Cancer in general
Glioma							
Ryan et al., 1992	Australia, Oceania	Case–control	Cases: -Glioma: 110 -Meningioma: 60 Controls: 417	25–74	ADELAIDE ADULT BRAIN TUMOR STUDY 1987–90	3	-Glioma -Meningioma
Hurley et al., 1996	Australia, Oceania	Case–control	Cases: 416 (40) Controls: 422 (40.3)	Cases: 48.9 (14.3) Controls: 50.2 (14.3)	Melbourne adult brain tumor study	4	Glioma cancer
Efird et al., 2004	USA, America	Cohort	142,085	25– > 65	KPMCP-NC	21 years; Mean 13.2 ± 6.7	Glioma cancer
Baglietto et al., 2010	Australia, Oceania	Cohort	41,514 (59)	27–81	Melbourne Collaborative Cohort Study	15	Glioblastoma

* Age: in mean (SD) or range as reported in the studies. USA, United States; NA, Not applicable; NR, Not reported; NBSS, Canadian National Breast Screening Study; EPIC, European Prospective Investigation into Cancer and Nutrition; NLCS, The Netherlands Cohort Study; NHEFS, Epidemiologic Follow-up Study; PROMEN STUDY, study of prostate cancer and hormones and alcohol intake; NIH-AARP, National Institutes of Health-AARP Diet and Health Study; CTS, California Teachers Study; OVAL-BC, Ovarian Cancer in Alberta and British Columbia; EAC, Esophageal adenocarcinoma; EGJAG, Esophagogastric junction adenocarcinoma; ESCC, Esophageal squamous cell carcinoma; NHS, Nurses’ Health Study; HPFS, Health Professionals Follow-up Study; BCC, Basal cell carcinoma; SCC, Squamous cell carcinoma; NCCCS-II, The North Carolina Colon Cancer Study-Phase II; KPMCP-NC, Kaiser Permanente Medical Care Program of Northern California.

The references presented in this table are available in Supplementary material.

The Newcastle-Ottawa Quality Assessment Scale was used to assess the risk of bias for longitudinal studies. This tool consists of eight items divided into three categories: (i) selection, (ii) comparability, and (iii) exposure or outcome (depending on whether it is a case–control or cohort study, respectively). Each study is eligible to receive four stars for selection, three stars for exposure, and two stars for comparability. Cohort studies can receive a maximum score of nine, whereas case–control studies can receive a maximum of eight stars, as case–control studies can score with a maximum of one star in the comparability category. A higher score on this scale indicates a lower risk of bias (25).

Study selection, data extraction, and risk of bias assessments were performed by two independent reviewers (ML-LT and CA-B). Disagreements were resolved by consensus or with the intervention of a third investigator (IC-R).

### Statistical analysis and data synthesis

A meta-analysis was conducted to analyze the association between wine consumption and the risk of developing different types of cancer after classifying studies according to the reported cancer type. When two studies included data from the same sample, we included the study with the larger sample size in the meta-analysis. A 5-year follow-up of the included studies was set as an inclusion criterion for the meta-analysis to ensure data quality. Regarding gynecological cancers, we studied breast and ovarian cancer separately since they are influenced by different risk factors and have different prevalence rates; breast cancer is more prevalent than ovarian cancer (17). The reported relative risk (RR) and odds ratios (OR) were both included in the meta-analysis (26). However, when studies reported the hazard ratio (HR), it was converted to RR using the following formula: RR = (1 -eHRln(1 – r))/r (26).

The DerSimonian and Laird random-effects (27) models were used to calculate the pooled RR and its 95% confidence intervals (CIs) for each type of cancer. The Cochrane Handbook recommendations were used to examine inconsistency, with ranges from 0 to 100% (28). According to the I2, inconsistency was considered unimportant (0–30%), moderate (≥30–50%), substantial (≥50–75%), or considerable (≥75–100%). Corresponding *p* values were considered. In addition, the τ2 test was used to assess heterogeneity and was interpreted as low when it was below 0.04, moderate when it ranged from ≥0.04 to 0.14, and substantial when it ranged from ≥0.14 to 0.40 (29).

A sensitivity analysis was performed to assess the robustness of the summary estimates, eliminating each study one by one from the pooled estimates. Subgroup analyses were performed according to the continent. Random effects meta-regression analyses were used to address whether participants’ mean age, follow-up and percentage of female, whenever possible, as continuous variables, of wine exposure could modify the association of wine consumption and different types of cancer (ovarian, breast, colorectal and renal cancer). Finally, publication bias was assessed using Egger’s regression asymmetry test (30), where a *p* value of <0.10 determined whether significant publication bias existed.

All statistical analyses were conducted with STATA SE software, version 15 (StataCorp, College Station, TX, United States).

## Results

### Systematic review

**Figure 1:**
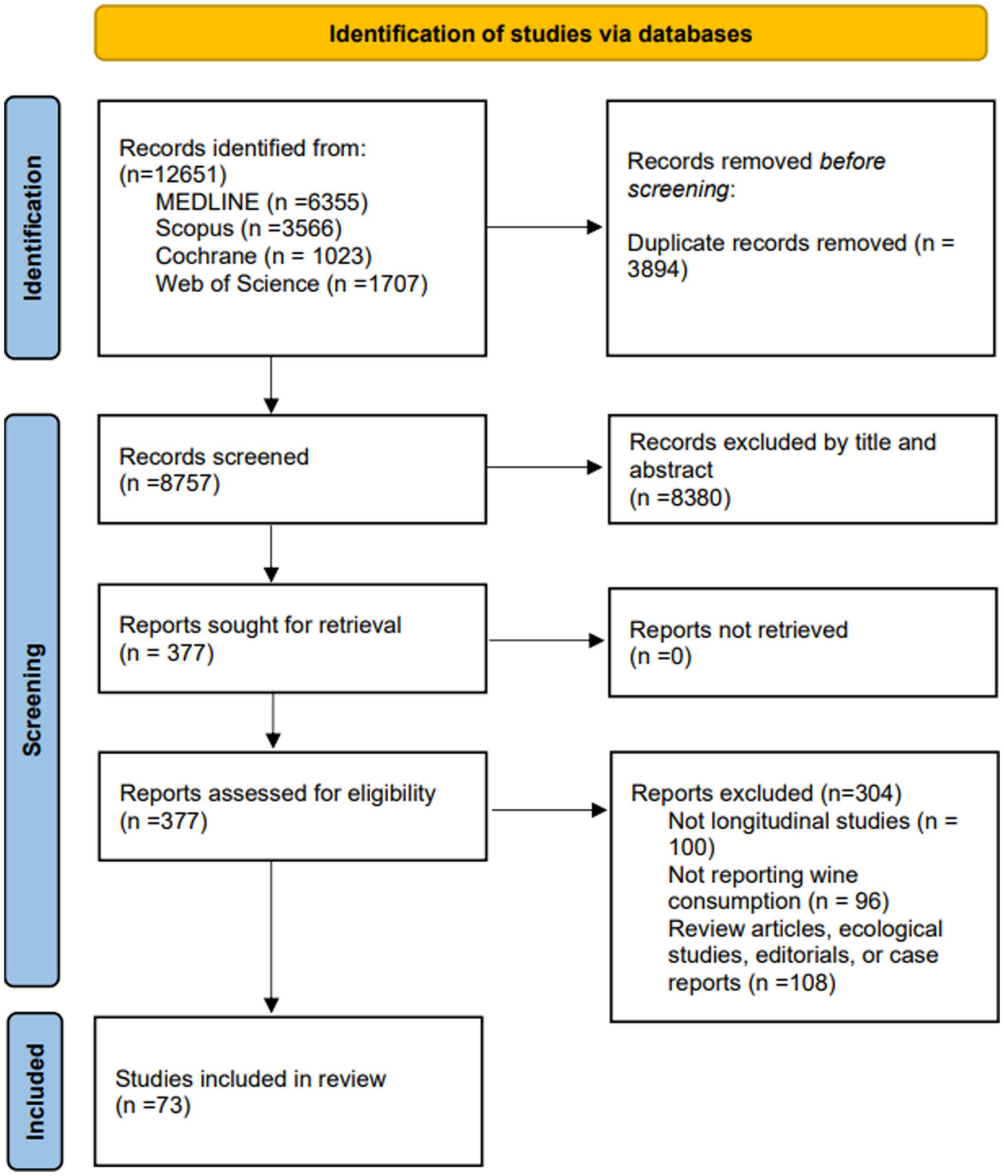
PRISMA 2020 flow diagram for new systematic reviews including database searches.

The literature search retrieved 12,651 studies; 8,380 were excluded in the title and abstract review, and 377 were selected for the eligibility assessment. From those, 73 (31–103) studies were included in the systematic review and 26 (31, 34–37, 41, 42, 44, 48, 50, 52, 56, 59, 67, 72, 74, 77, 79, 87–90, 92, 96, 102, 103) in the meta-analysis (Figure 1). The reasons for excluding studies are specified in the flow chart and Supplementary Table S2.

Of the included studies, 31 (31–61) were cohort studies, and 42 (62–103) were case–control studies. The studies were conducted in 19 different countries: the USA (30) (31, 34, 36, 38–41, 43, 45, 48, 49, 53, 55, 56, 58, 59, 62, 63, 67, 68, 75, 76, 78, 81, 82, 92, 94, 98, 99, 102), New Zealand (1) (71), Australia (8) (51, 57, 64, 70, 73, 80, 93, 101), Canada (8) (37, 60, 70, 74, 86, 89, 97, 103), Greece (2) (50, 70), Argentina (1) (70), Uruguay (2) (79, 85), Italy (11) (50, 65, 66, 69, 70, 72, 77, 84, 87, 88, 96), Netherlands (6) (32, 35, 44, 50, 52, 54), Hawaii (1) (90), Denmark (4) (33, 42, 46, 50), Puerto Rico (1) (91), the United Kingdom (2) (50, 61), Germany (1) (50), France (1) (50), Sweden (5) (47, 50, 83, 95, 100), Spain (1) (50), and Norway (1) (50). The studies were published between 1986 and 2021 with a total sample of 4,346,504 subjects aged between 18 and 103 years. The follow-up period reported by the cohort studies ranged from 2 (41) to 24 years (58). The studies were classified by the type of cancer reported; 11 studies reported information on breast cancer (36, 39, 41, 50, 51, 56, 63, 70, 71, 74, 78), 14 on prostate cancer (34, 35, 38, 45, 46, 77, 79, 82, 83, 86, 94–96, 98), four on renal cell cancer (47, 59, 88, 100), five on pancreatic cancer (39, 54, 55, 58, 84), 10 on ovarian cancer (44, 48, 62, 72, 87, 90, 92, 93, 93, 103), eight on upper airway cancers (33, 46, 65, 66, 69, 85, 91, 101), two on skin cancer (40, 51), one on lung cancer (97), 11 on colorectal cancers (31, 32, 42, 52, 64, 67, 68, 75, 76, 89, 102), and four on glioma (43). The studies reported wine consumption differently (31–63, 65–82, 84–103); 71 studies reported frequency and amount of consumption; four studies reported on whether wine was consumed or not (64, 73, 80, 83). In addition, 15 reported on consumption in oz (31, 36–39, 67, 78, 81, 82, 84, 90–92, 94, 98); 32 studies reported consumption in grams of ethanol (31, 35, 39, 40, 44, 45, 47–50, 52–54, 57, 59, 62, 63, 68, 74–76, 78, 84, 86, 87, 93, 95, 96, 99–102), 18 studies reported wine consumption in milliliters (32, 54, 55, 65, 66, 69, 72, 77, 79, 82, 84, 85, 87, 90, 91, 95, 96, 100); one study reported in standard drinking unit (51) and one study reported the % of ethanol (70). Information on the included studies is shown in Table 1. Finally, a different set of covariates was used to adjust the analyses reported by the included studies (Supplementary Table S3).

### Risk of bias assessment

The Newcastle-Ottawa Quality Assessment Scale was used to assess the risk of bias of the cohort studies. The total score of the included studies ranged from seven to nine stars. Only four studies did not have the highest score in the selection category (32, 40, 48, 59). In the comparability category, all studies scored the highest. Finally, in the outcome category there were four studies with one star (39, 47, 48, 61), 16 studies with two stars (31, 32, 38, 40–44, 48, 50–55, 58, 60), and the rest with three stars (33–37, 45, 46, 56, 57, 59) (Supplementary Table S4).

The Newcastle-Ottawa Quality Assessment Scale was used to assess the risk of bias of the case–control studies. The total score of the included studies ranged from six to eight stars. In the selection category, one study scored two stars (66), nine studies scored three stars (69, 70, 72, 84, 85, 88, 89, 96, 103), and the remaining studies scored the maximum (62–65, 67, 68, 71, 73–83, 86, 87, 90–95, 97, 102). In the comparability category, all studies scored the highest. Finally, in the outcome category, 10 studies (65, 67, 70, 89, 93, 95, 99, 100, 101, 103) scored one star and the remaining included studies scored two stars (62–64, 66, 68, 69, 71–88, 90–92, 94, 96–98, 102) (Supplementary Table S5).

### Meta-analysis

#### Association between wine consumption and breast and ovarian cancer

**Figure 2:**
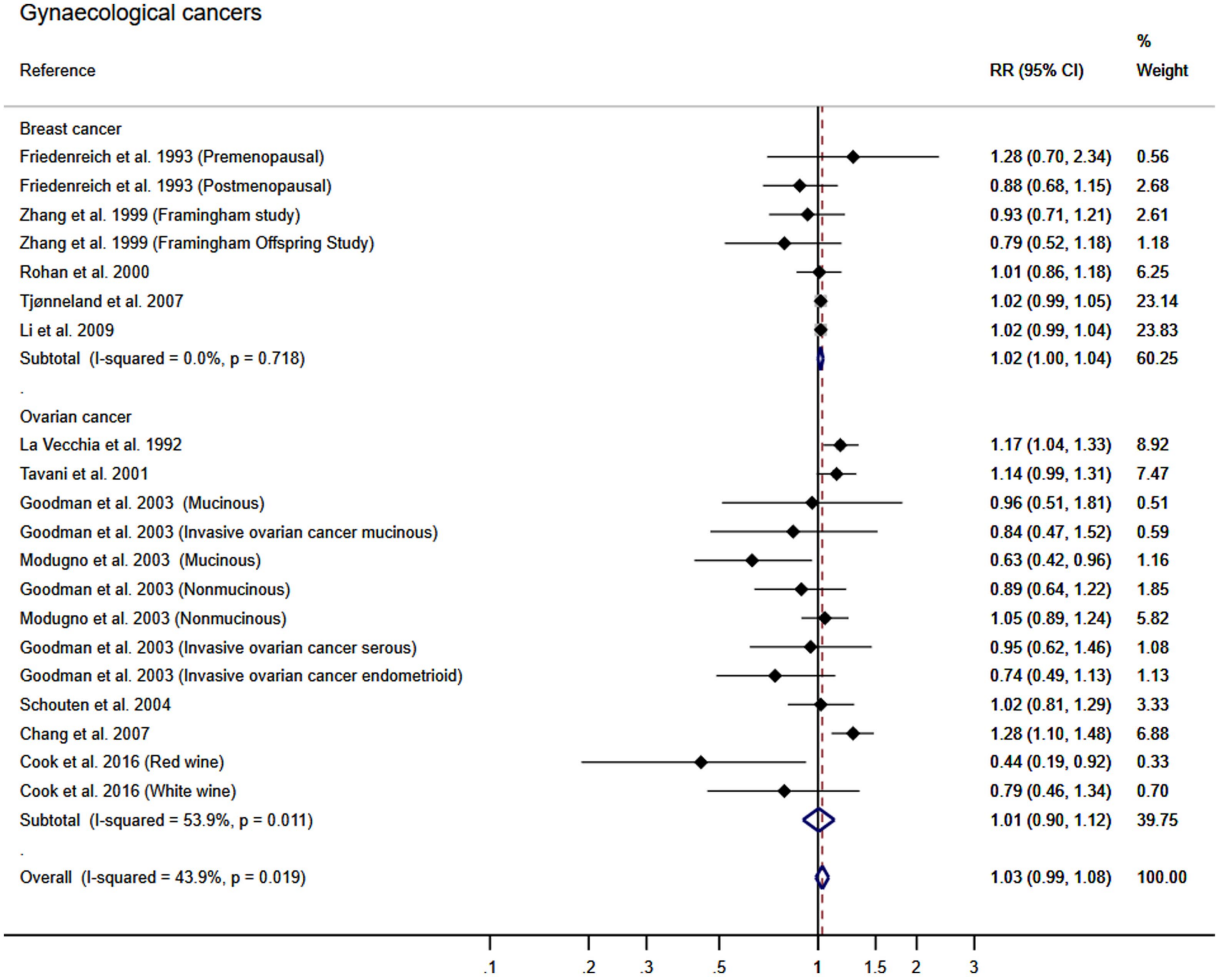
Meta-analysis of the association between wine consumption and gynecological cancers. Horizontal lines represent the 95% confidence intervals of the study, and the black boxes represent the effect size of each study.

**Figure 3:**
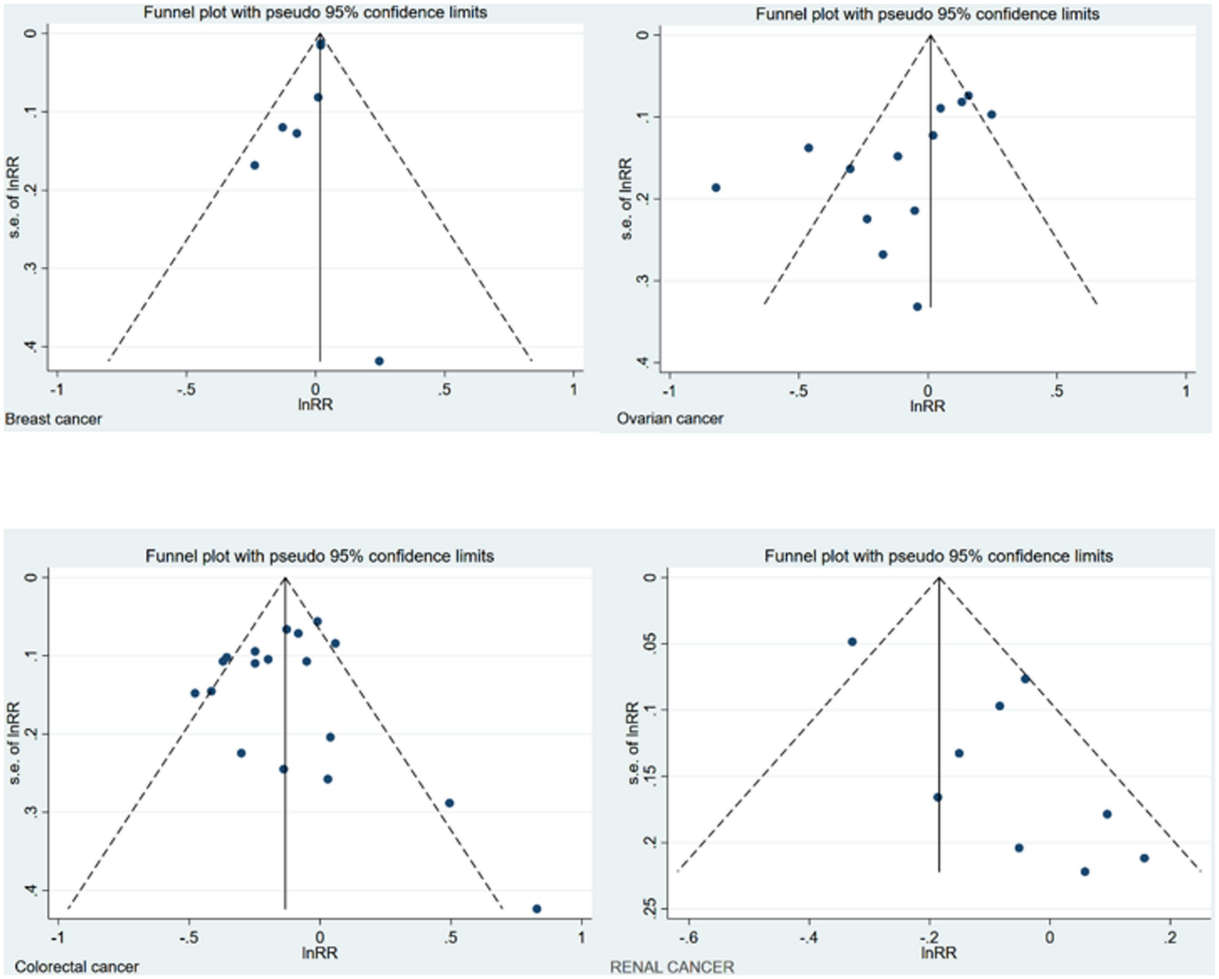
Funnel plot of the different cancers.

Using the DerSimonian and Laird random-effects models, the pooled RR for gynecological cancers was 1.03 (95% CI: 0.99, 1.08; *I*^2^: 43.9% τ^2^: 0.0021). Furthermore, the pooled RR for the association between wine consumption and the development of breast cancer was 1.02 (95% CI: 1.00, 1.04). That of ovarian cancer was 1.01 (95% CI: 0.90, 1.12). The heterogeneity of these estimates was unimportant for breast cancer and substantial for ovarian cancer (*I*^2^: 0% τ^2^: 0.0000; and *I*^2^: 54% τ^2^: 0.0173, respectively) (Figure 2). Finally, no publication bias for breast was found using Egger’s test, but publication bias was found for ovarian cancer (*p* = 0.141, *p* = 0.021; respectively). The asymmetry in the funnel plot was confirmed for ovarian cancer (Figure 3).

#### Association between wine consumption and colorectal cancer

**Figure 4:**
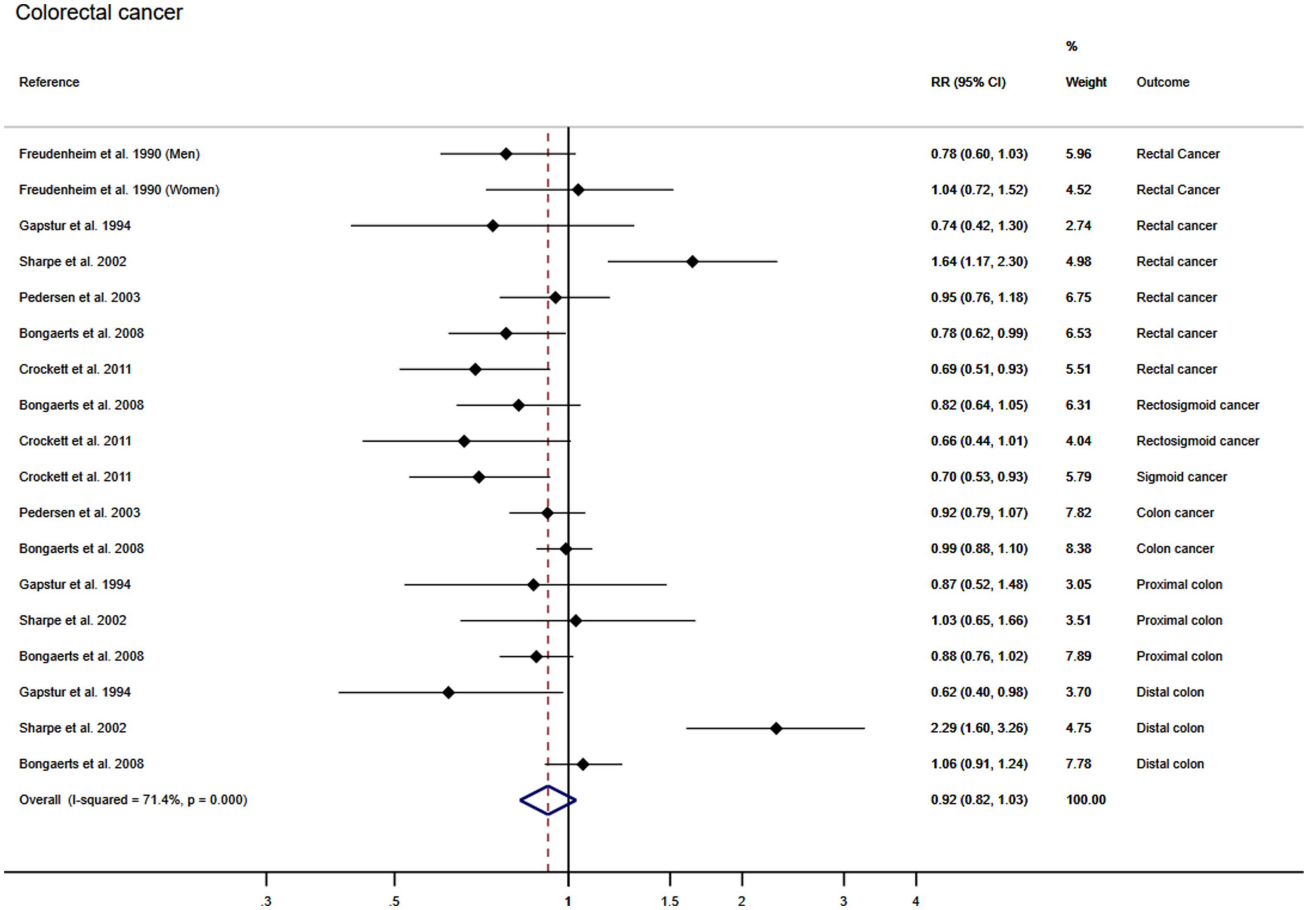
Meta-analysis of the association between wine consumption and colorectal cancer. Horizontal lines represent the 95% confidence intervals of the study, and the black boxes represent the effect size of each study.

Using the DerSimonian and Laird random effect models, the pooled RR for the association between wine consumption and the colorectal cancer was 0.92 (95% CI: 0.82, 1.03). The heterogeneity of these estimates was substantial (*I*^2^: 71.4% τ^2^: 0.0356) (Figure 4). Finally, publication bias was not found through Egger’s test for the association between wine consumption and colorectal (*p* = 0.884) cancer.

#### Association between wine consumption and renal cancer

**Figure 5:**
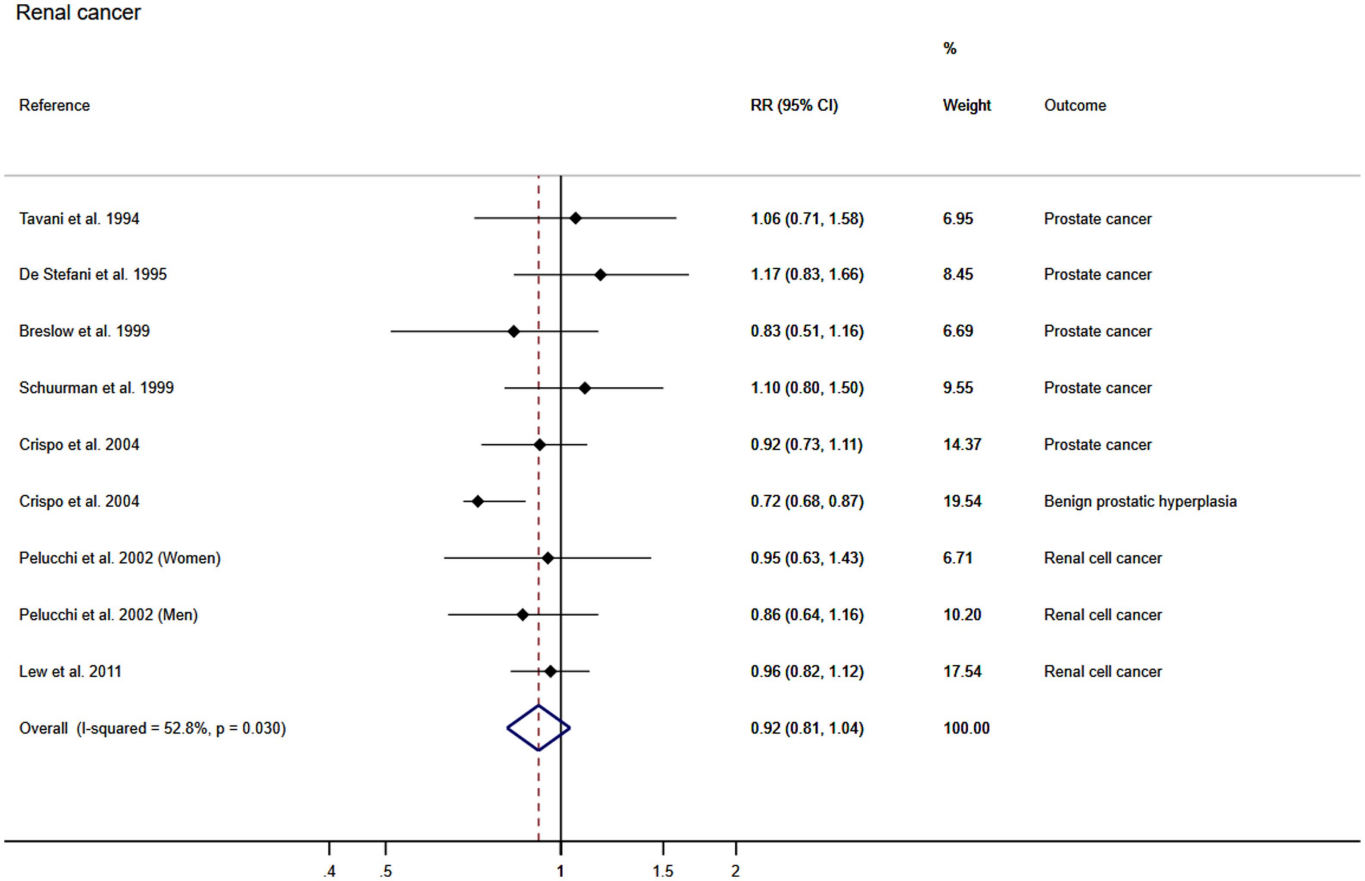
Meta-analysis of the association between wine consumption and renal cancer. Horizontal lines represent the 95% confidence intervals of the study, and the black boxes represent the effect size of each study.

Using the DerSimonian and Laird random-effects models, the pooled RR for the association between wine consumption and the development of renal cancer was 0.92 (95% CI: 0.81, 1.04). The heterogeneity of this estimate was substantial (*I*^2^: 52.8% τ^2^: 0.0169) (Figure 5). Finally, publication bias was found through Egger’s test for the association between wine consumption and renal cancer (*p* = 0.021) and the funnel plot presented the asymmetry (Figure 3).

### Association between wine consumption and pancreatic, skin, lung, brain, upper digestive tract and general cancer

**Figure 6:**
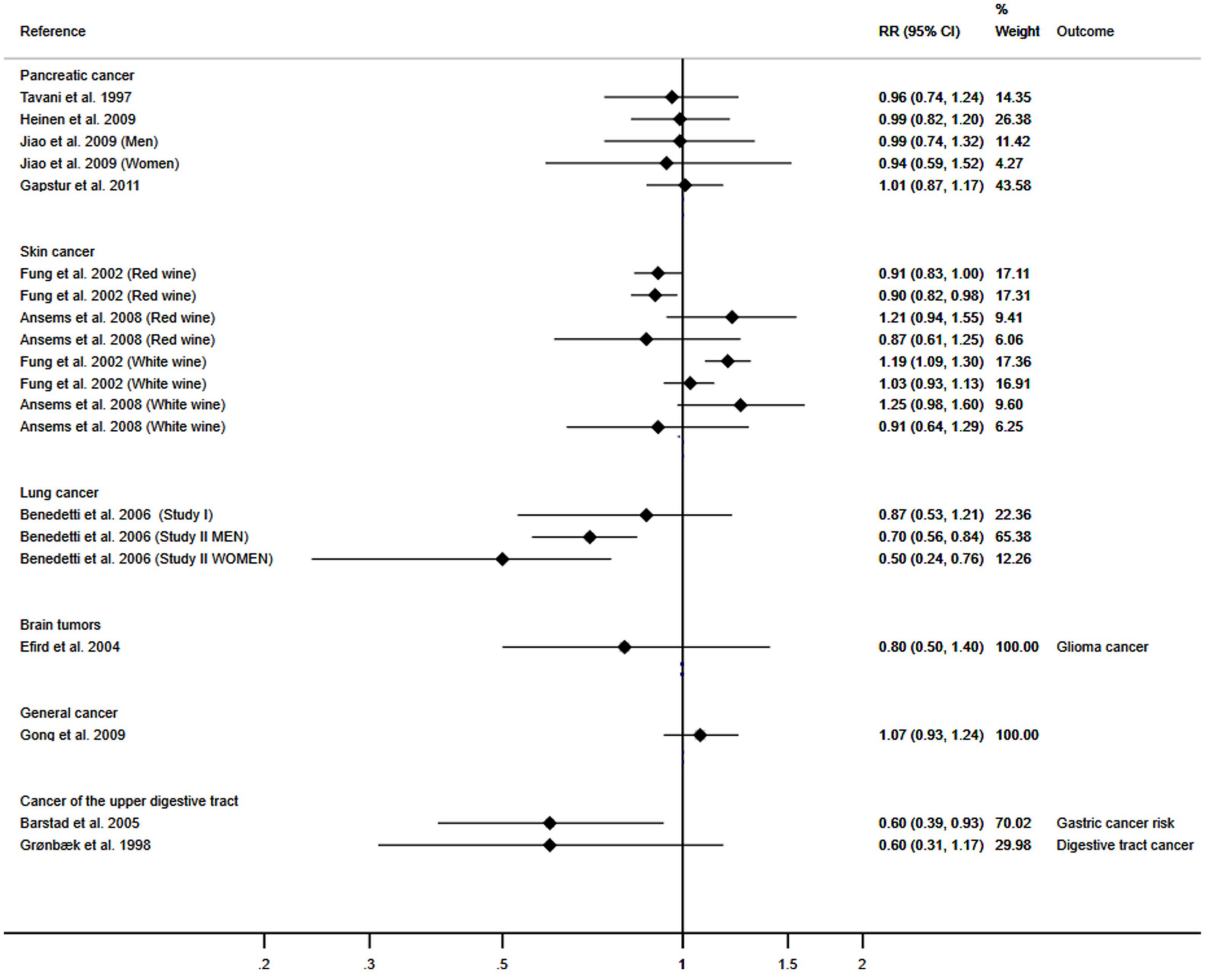
Meta-analysis of the association between wine consumption and pancreatic, skin, lung, brain and general cancer. Horizontal lines represent the 95% confidence intervals of the study, and the black boxes represent the effect size of each study.

The number of included studies focusing on these cancers was insufficient to perform a meta-analysis; therefore, a graphical representation is shown. The trend demonstrates a protective association between wine consumption and the development of pancreatic, skin, lung, brain, upper digestive tract and general cancer (Figure 6).

### Sensitivity analysis

The pooled RR estimations for the association of wine consumption with the development of different types of cancer were not significantly modified (in magnitude or direction) when data from individual studies were removed from the analysis one by one (Supplementary Table S6).

### Subgroup analysis and meta-regression models

When subgroup analyses were performed according to the continent where studies were conducted, the pooled RR estimate showed no significant differences for the different types of cancer (Supplementary Table S7). Random-effects meta-regression models showed that follow up could influence the pooled RR estimate for the association between wine consumption and renal cancer (*p* = 0.048) and colorectal cancer (*p* = 0.023) (Supplementary Table S8).

## Discussion

The aim of this study was to assess the association between wine consumption and the development of different types of cancer. Meta-regressions were performed to determine whether the association of wine with different types of cancer could be modified by individual and study characteristics including follow-up time, percentage of women, and mean age of participants. Subgroup analyses were performed to depict whether there were differences between continents. Our results indicate no association between wine consumption and the development of general cancer or upper digestive tract, colorectal, renal, pancreatic, skin, lung, brain, and gynecological cancer. Furthermore, no differences were observed after subgroup analyses and meta-regression showed that follow-up could only influence the association between wine consumption and renal and colorectal cancer.

No association was found between wine consumption and the risk of developing gynecological cancer. Evidence has suggested that the greater the amount of alcohol consumption is, the greater the risk of developing breast cancer will be (104); the risk of breast cancer has shown to be increased two-fold among premenopausal women engaging in high alcohol consumption (36), and this risk has also been observed among early consumers of beer and spirits. However, other studies have reported no positive or negative association between wine consumption and breast cancer (36, 37, 63), and additional studies have demonstrated a protective effect of red wine consumption during adolescence and early adulthood on mammographic density (105). Mammographic density benefits have also been associated with white wine consumption (106) among postmenopausal women (107). In addition, our data do not show any association between wine consumption and ovarian cancer risk. Previous evidence has suggested an inverse association between wine consumption and the risk of developing ovarian cancer (90, 93), which is stronger for red wine (103). The possible inverse association could be due to the specific components of wine, such as antioxidants and/or phytoestrogens. In the case of breast cancer, this is a chemo-preventive effect that reduces tumor methylation (108).

Our analyses revealed no association between wine consumption and the risk of developing renal cancer, although previous evidence has shown that moderate wine intake is associated with a lower risk of renal cancer. This protective trend has been observed for both red and white wine (100), and has been found to be stronger among postmenopausal women (47). In the case of prostate cancer, evidence suggests that red wine consumption is associated with decreased risk, particularly regarding aggressive prostate cancer. Our findings did not confirm these assertions.

Gastric cancer has been associated with alcohol consumption for many years, and this association has been reported to be weaker for wine drinkers than for drinkers of other alcoholic beverages (109–111). This observation may be due to the effect of wine on *Helicobacter pylori*, which is associated with gastric cancer; additionally, the alcohol in wine may increase gastric acidity, which could prevent the growth of different bacterial species (112). Unlike other alcoholic beverages, a moderate daily intake of wine appears to prevent the development of gastric and esophageal cancer (46, 101); however, excessive wine consumption and the consumption of other alcoholic beverages increases this risk (100). Similarly, alcohol consumption has been associated with colon and rectum cancer; however, previous studies have reported an inverse or null relationship between wine consumption and the risk of developing cancer of the colon and rectum (14, 31, 89, 113, 114).

Our results revealed no association between wine consumption and lung cancer. The association between wine consumption and lung cancer has been described as J-shaped, with moderate daily wine drinkers having a lower risk of developing this type of cancer than nondrinkers and heavy drinkers (97). In addition, regarding lung cancer development, it has been shown that there is a concomitant effect of smoking and alcoholic beverage consumption, excluding wine, especially among men (97). However, alcohol consumption has been associated with multiple pathologies, such as pancreatitis. Although pancreatitis is a risk factor for pancreatic cancer (115), alcohol (116) or wine consumption has not been directly linked to pancreatic cancer.

We could not determine the association between wine consumption and the risk of developing other types of cancer. Regarding skin cancer, basal cell carcinoma is the most common skin cancer in the fair-skinned population, and its incidence has increased over the last 20 years, with fair skin, hair and eye color being risk factors owing to an increased susceptibility to sunburn (117, 118). Existing evidence shows an inverse association between red wine consumption and skin cancer only in women (40). Regarding brain cancer, alcohol consumption has been shown to be a risk factor due to the ability of alcohol to cross the blood–brain barrier (57, 119, 120). However, a previous meta-analysis demonstrated that this might not be the case for wine, as light wine consumption could prevent cognitive impairment due to the neuroprotective effects of wine components (121, 122).

Many components in wine could have anticarcinogenic effects, such as resveratrol, which plays antioxidant, antimutagenic, and anti-inflammatory roles in carcinogenesis (123). The anti-inflammatory role of resveratrol is found in both acute and chronic phases of the inflammatory process (123). In addition, resveratrol is responsible for inhibiting the cellular process of tumor initiation, promotion, and progression by inhibiting the COX-1 cyclooxygenase activity involved in antitumour promotion (123). Resveratrol provides all these benefits mainly in its trans isoform, which can only be found in peanuts and wine. Resveratrol levels can vary across different wine types depending on the fermentation length. In addition, resveratrol levels are lower in white wine than in red wine because the skins of white grapes are removed before fermentation (124). Other components with anticarcinogenic properties are anthocyanins, quercetin, and tannins, all of which have been shown to protect against ultraviolet radiation, acting on free radicals, suppressing the activity of cyclooxygenase-2 (COX-2), and acting against the enzyme myeloperoxidase, thus preventing the development of skin cancer (125–127).

The analyses included in this meta-analysis are adjusted by different covariables, many of them related to healthy behaviors. A healthy dietary pattern is associated with a lower incidence of cardiometabolic events, including wine consumption (128), possibly because alcoholic beverage preference is related to socioeconomic status, lifestyle, and diet (129). In comparison with other alcoholic beverages (129), wine consumption is associated with healthier lifestyles, increased physical activity (130), and less smoking (131). Our analyses could not determine whether these factors influenced the relationship between wine consumption and cancer development. Moreover, the combination of all these factors could be the reason for the greater health benefits (129).

This systematic review and meta-analysis has some limitations that should be mentioned. First, while the WHO sets light-moderate consumption at 20 grams of ethanol per day for men and 10 grams of ethanol per day for women, there is no common definition of wine consumption across studies, the included studies differed in the methods used to measure wine consumption and did not report the specific volume of wine consumed. Reporting the alcohol consumption using the same units is needed to properly determine its effect in different populations. The lack of globally agreed recommendations or safe drinking limits could be a limitation in addressing this issue. Second, the literature search was conducted in English and Spanish and did not include gray literature, which may have missed some potential articles for our study. Thirdly, after assessing the risk of bias in the cohort studies, it was found that many of the studies did not provide a proper outcome assessment, as they were often self-reported by the participants, thus increasing the risk of bias. Regarding the risk of bias in case–control studies, there were studies that selected controls from hospital samples and there were also studies that did not assess the outcome correctly, either because it was not blinded or because it was self-reported, these reasons could decrease the quality of our study. Fourth, meta-regressions by relevant participant characteristics, including the percentage of women, the mean age, or the follow-up time, could not be performed in certain types of cancer due to the lack of data, this could influence the quality of our results as we cannot depict whether the association of wine with certain cancers could be modified by characteristics of the participants or of the study. Fifth, our results may be influenced by confounding variables such as diet, socioeconomic status, and lifestyle although the most covariate-adjusted analyses were selected to try to avoid the influence of other covariates. Sixth, the heterogeneity of the confounding factors adjusted for in each study must be considered a limitation. Finally, due to a lack of data, the association between wine consumption and the development of different cancers could not be analyzed by type of wine or sex, it would be interesting to know which type of wine provides the greatest benefits and whether there is a difference in these benefits according to gender.

In summary, this systematic review and meta-analysis revealed no association between wine consumption and general, upper digestive tract, colorectal, renal, pancreatic, skin, lung, brain, and gynecological cancers. Caution should always be exercised in populations most vulnerable to alcohol consumption or those with pathologies. Finally, more research is needed to evaluate wine consumption independently from that of other alcoholic beverages, and guidelines for safe wine consumption should be included in health recommendations.

## Data availability statement

The original contributions presented in the study are included in the article/Supplementary material, further inquiries can be directed to the corresponding author.

## Author contributions

ML-L-T and CÁ-B: conceptualization, investigation, and writing—original draft preparation. ML-L-T, CÁ-B, and IC-R: methodology. IC-R and CÁ-B: software. CP-M and BB-P: validation and visualization. ML-L-T and IC-R: formal analysis. ML-L-T, BB-P, and CP-M: resources. CÁ-B and VM-V: data curation. VM-V: writing—review and editing. CÁ-B: supervision. All authors revised and approved the final version of the articles.

## Funding

This research was funded by FEDER funds. ML-L-T was supported by a grant from the University of Castilla-La Mancha (2022-PROD-20657). BB-P was supported by a grant from the University of Castilla-La Mancha, Spain, co-financed by the European Social Fund (2020-PREDUCLM-16746). CP-M was supported by a grant from the Universidad de Castilla-La Mancha (2018-CPUCLM-7939).

## Conflict of interest

The authors declare that the research was conducted in the absence of any commercial or financial relationships that could be construed as a potential conflict of interest.

## Publisher’s note

All claims expressed in this article are solely those of the authors and do not necessarily represent those of their affiliated organizations, or those of the publisher, the editors and the reviewers. Any product that may be evaluated in this article, or claim that may be made by its manufacturer, is not guaranteed or endorsed by the publisher.
