# Supplementary material for: Association between wine consumption and cancer: a systematic review and meta-analysis
Source: Front Nutr. 2023 Sep 4;10:1197745. doi: 10.3389/fnut.2023.1197745 (PMC10507274; doi:10.3389/fnut.2023.1197745)
Supplement: Supplementary file 1 [file Data_Sheet_1.docx]

**SUPPLEMENTARY MATERIAL**

**TABLE S1.** Complete search strategy for MEDLINE.

| Adults  OR  “Young adults”  OR  “adults populations”  OR  “adults subjects”  OR  older  OR  elderly  OR  “elderly people”  OR  “older people” | AND | Alcohol  OR  Wine  OR  “Alcohol consumption”  OR  “Wine consumption” | AND | neoplasm  OR  cancer  OR  tumor  OR  “cancer risk”  OR  carcinogen*  OR  mortalit* | AND | Cohort  OR  “Case-control”  OR  “Longitudinal studies”  OR  “Prospective studies” |
| --- | --- | --- | --- | --- | --- | --- |

| **Reference** | **Reason for exclusion** |
| --- | --- |
| Michaud et al. 2010 | Review articles, ecological studies, editorials, or case reports |
| Genkinger et al. 2006 | Review articles, ecological studies, editorials, or case reports |
| Genkinger et al. 2009 | Review articles, ecological studies, editorials, or case reports |
| Lucenteforte et al. 2012 | Review articles, ecological studies, editorials, or case reports |
| Jung et al. 2012 | Nor reporting wine consumption |
| Lowry et al. 2016 | Not reporting wine consumption |
| Kune et al. 1992 | Not longitudinal studies |
| Larsson et al. 2004 | Not reporting wine consumption |
| Liu et al. 2015 | Review articles, ecological studies, editorials, or case reports |
| Longnecker et al. 1990 | Review articles, ecological studies, editorials, or case reports |
| Magnusson et al. 2007 | Not reporting wine consumption |
| Nelson et al. 2013 | Not reporting wine consumption |
| Pang et al. 2017 | Not longitudinal studies |
| Schouten et al. 2016 | Not reporting wine consumption |
| Smith et al. 1989 | Not longitudinal studies |
| Shufelt et al. 2012 | Not longitudinal studies |
| Viner et al. 2018 | Not reporting wine consumption |
| Larsson et al. 2004 | Review articles, ecological studies, editorials, or case reports |
| Allen et al. 2009 | Not reporting wine consumption |
| Smith-Warner et al. 1998 | Review articles, ecological studies, editorials, or case reports |
| Lee et al. 2007 | Review articles, ecological studies, editorials, or case reports |
| Cho et al. 2004 | Review articles, ecological studies, editorials, or case reports |
| Ferrari et al. 2007 | Review articles, ecological studies, editorials, or case reports |
| Zhang et al. 2021 | Not reporting wine consumption |
| Bonnin-Scaon et al. 2002 | Not reporting wine consumption |
| Hinds et al. 1980 | Not reporting wine consumption |
| Hoey et al. 1981 | Not longitudinal studies |
| Kono et al. 1979 | Not reporting wine consumption |
| Kune et al. 1987 | Not reporting wine consumption |
| Bagnardi et al. 2001 | Review articles, ecological studies, editorials, or case reports |
| Song et al. 2012 | Review articles, ecological studies, editorials, or case reports |
| Yan-Hong et al. 2015 | Review articles, ecological studies, editorials, or case reports |
| Bagnardi et al. 2001 | Review articles, ecological studies, editorials, or case reports |
| Dennis et al. 2000 | Review articles, ecological studies, editorials, or case reports |
| Golan et al. 2018 | Review articles, ecological studies, editorials, or case reports |
| Haseeb et al. 2017 | Review articles, ecological studies, editorials, or case reports |
| Kim et al. 2010 | Review articles, ecological studies, editorials, or case reports |
| Di Castelnuovo et al. 2002 | Review articles, ecological studies, editorials, or case reports |
| Costentin et al. 2018 | Not reporting wine consumption |
| Silva et al. 2020 | Review articles, ecological studies, editorials, or case reports |
| Amor et al. 2018 | Review articles, ecological studies, editorials, or case reports |
| Vartolomei et al. 2018 | Review articles, ecological studies, editorials, or case reports |
| Macke et al. 2022 | Review articles, ecological studies, editorials, or case reports |
| Minzer et al. 2020 | Review articles, ecological studies, editorials, or case reports |
| García-Lavandeira et al. 2016 | Review articles, ecological studies, editorials, or case reports |
| Sancho et al. 2014 | Review articles, ecological studies, editorials, or case reports |
| Jasinki et al. 2013 | Review articles, ecological studies, editorials, or case reports |
| Giacosa et al. 2012 | Review articles, ecological studies, editorials, or case reports |
| Bianchini et al. 2003 | Review articles, ecological studies, editorials, or case reports |
| Ferrer-Gallego et al. 2022 | Review articles, ecological studies, editorials, or case reports |
| Jang et al. 2022 | Review articles, ecological studies, editorials, or case reports |
| Laudisio et al. 2021 | Review articles, ecological studies, editorials, or case reports |
| Ferraz da Costa et al. 2020 | Review articles, ecological studies, editorials, or case reports |
| Das et al. 2021 | Review articles, ecological studies, editorials, or case reports |
| Day et al. 2020 | Review articles, ecological studies, editorials, or case reports |
| McMahon et al. 2020 | Review articles, ecological studies, editorials, or case reports |
| Farinetti et al. 2017 | Review articles, ecological studies, editorials, or case reports |
| Fernandes et al. 2017 | Review articles, ecological studies, editorials, or case reports |
| Kamaleddin et al. 2016 | Review articles, ecological studies, editorials, or case reports |
| Varoni et al. 2015 | Review articles, ecological studies, editorials, or case reports |
| Klarish et al. 2015 | Review articles, ecological studies, editorials, or case reports |
| Sancho et al. 2015 | Review articles, ecological studies, editorials, or case reports |
| Yang et al. 2014 | Review articles, ecological studies, editorials, or case reports |
| Arranz et al. 2012 | Review articles, ecological studies, editorials, or case reports |
| Muqbil et al.2012 | Review articles, ecological studies, editorials, or case reports |
| Rizos et al. 2010 | Review articles, ecological studies, editorials, or case reports |
| Kraft et al. 2009 | Review articles, ecological studies, editorials, or case reports |
| Hjartåker et al. 2010 | Review articles, ecological studies, editorials, or case reports |
| Chen et al. 2009 | Review articles, ecological studies, editorials, or case reports |
| Kamholz et al. 2006 | Review articles, ecological studies, editorials, or case reports |
| Mathé et al. 1999 | Review articles, ecological studies, editorials, or case reports |
| Choi et al. 1991 | Not reporting wine consumption |
| Bonnin-Scaon et al. 2002 | Not reporting wine consumption |
| Shufelt et al. 2012 | Not reporting wine consumption |
| Galeone et al. 2013 | Not longitudinal studies |
| Viner et al. 2019 | Not longitudinal studies |
| Fragopoulou et al. 2021 | Not longitudinal studies |
| Zhang et al. 2021 | Not reporting wine consumption |
| Petti et al. 2005 | Not reporting wine consumption |
| Harvey et al. 1987 | Not reporting wine consumption |
| Jatoi et al. 2016 | Not longitudinal studies |
| Ansems et al. 2008 | Not longitudinal studies |
| Ranstam et al. 1995 | Not reporting wine consumption |
| Lowry et al. 2016 | Not reporting wine consumption |
| Rosenberg et al. 1993 | Review articles, ecological studies, editorials, or case reports |
| Van de Brandt et al. 1995 | Not reporting wine consumption |
| Holmberg et al. 1994 | Not reporting wine consumption |
| Graham et al. 1992 | Review articles, ecological studies, editorials, or case reports |
| Mills et al. 1989 | Not reporting wine consumption |
| Bagnardi et al. 2001 | Not longitudinal studies |
| Benedetti et al. 2009 | Not reporting wine consumption |
| Brownson et al. 1988 | Not reporting wine consumption |
| Di Castelnuovo et al. 2006 | Review articles, ecological studies, editorials, or case reports |
| Goodman et al. 1986 | Not reporting wine consumption |
| Hu et al. 2008 | Not longitudinal studies |
| Kan et al. 2011 | Not longitudinal studies |
| Kim et al. 2000 | Review articles, ecological studies, editorials, or case reports |
| Kreiger et al. 1993 | Not reporting wine consumption |
| Lee et al. 2007 | Not reporting wine consumption |
| McLaughlin et al. 1984 | Not reporting wine consumption |
| Parker et al. 2010 | Not reporting wine consumption |
| Wolk et al. 1996 | Review articles, ecological studies, editorials, or case reports |
| Kelsey et al. 1993 | Not longitudinal studies |
| Hunter et al. 1996 | Not reporting wine consumption |
| Holmberg et al. 1994 | Not longitudinal studies |
| Mills et al. 1989 | Not reporting wine consumption |
| Willet et al. 1987 | Not reporting wine consumption |
| Holmberg et al. 1995 | Not reporting wine consumption |
| Schatzkin et al. 1989 | Not longitudinal studies |
| Hiatt et al. 1984 | Not reporting wine consumption |
| Le et al. 1984 | Not longitudinal studies |
| Levi et al. 1993 | Not reporting wine consumption |
| Martín-Moreno et al. 1993 | Not reporting wine consumption |
| Katsouyanni et al. 1994 | Not reporting wine consumption |
| Landa et al. 1994 | Not reporting wine consumption |
| Levi et al. 1996 | Not reporting wine consumption |
| Viel et al. 1997 | Not longitudinal studies |
| Bowlin et al. 1997 | Not reporting wine consumption |
| Katsouyanni et al. 1986 | Not reporting wine consumption |
| Adami et al. 1988 | Not longitudinal studies |
| Ewertz et al. 1991 | Not reporting wine consumption |
| Garro et al. 1990 | Not longitudinal studies |
| O’Connell et al. 1987 | Not longitudinal studies |
| Hiatt et al. 1988 | Not longitudinal studies |
| Parker et al. 2002 | Not reporting wine consumption |
| Brownson et al. 1988 | Not reporting wine consumption |
| Talamini et al. 1990 | Not reporting wine consumption |
| Maclure et al. 1990 | Not reporting wine consumption |
| Benhamou et al. 1993 | Not reporting wine consumption |
| Lindblad et al. 1997 | Not reporting wine consumption |
| Mehabir et al. 2005 | Not reporting wine consumption |
| Bandera et al. 1997 | Not reporting wine consumption |
| Flood et al. 2002 | Not reporting wine consumption |
| Calle et al. 2002 | Not longitudinal studies |
| Adami et al. 1992 | Not reporting wine consumption |
| Glynn et al. 1996 | Not reporting wine consumption |
| Giovannucci et al. 1995 | Not reporting wine consumption |
| Bandera et al. 2002 | Not reporting wine consumption |
| Jain et al. 1996 | Not reporting wine consumption |
| Munger et al. 1992 | Review articles, ecological studies, editorials, or case reports |
| Giovannucci et al. 1991 | Review articles, ecological studies, editorials, or case reports |
| Bostick et al. 1993 | Review articles, ecological studies, editorials, or case reports |
| Kampman et al. 1994 | Not reporting wine consumption |
| Pietinen et al. 1999 | Not longitudinal studies |
| Frezza et al. 1990 | Not longitudinal studies |
| Bruzzi et al. 1985 | Review articles, ecological studies, editorials, or case reports |
| Le Marchand et al. 1997 | Not reporting wine consumption |
| Seitz et al. 1990 | Not longitudinal studies |
| Choi et al. 1999 | Review articles, ecological studies, editorials, or case reports |
| Kune et al. 1991 | Review articles, ecological studies, editorials, or case reports |
| Williams et al. 1977 | Not longitudinal studies |
| Pollack et al. 1984 | Not reporting wine consumption |
| Hirayama et al. 1990 | Not reporting wine consumption |
| Stemmermann et al. 1990 | Not reporting wine consumption |
| Hu et al. 1991 | Not reporting wine consumption |
| Chyou et al. 1996 | Not reporting wine consumption |
| Tuyns et al. 1982 | Not longitudinal studies |
| Kune et al. 1986 | Not reporting wine consumption |
| Peters et al. 1989 | Not reporting wine consumption |
| Ferraroni et al. 1989 | Review articles, ecological studies, editorials, or case reports |
| Peters et al. 1992 | Review articles, ecological studies, editorials, or case reports |
| Slattery et al. 1990 | Review articles, ecological studies, editorials, or case reports |
| Slattery et al. 1997 | Review articles, ecological studies, editorials, or case reports |
| Bidoli et al. 1992 | Not reporting wine consumption |
| Breslow et al. 1974 | Review articles, ecological studies, editorials, or case reports |
| Riboli et al. 1991 | Not longitudinal studies |
| Terry et al. 2003 | Not reporting wine consumption |
| Tzonou et al. 1984 | Not reporting wine consumption |
| Webb et al. 2004 | Not longitudinal studies |
| Sand et al. 2007 | Not longitudinal studies |
| Lowenfels et al. 2006 | Not longitudinal studies |
| Lowenfels et al. 2005 | Not longitudinal studies |
| Bouchardy et al. 1990 | Not longitudinal studies |
| Bueno de Mesquita et al.1992 | Not reporting wine consumption |
| Clavel et al. 1989 | Not reporting wine consumption |
| Ferraroni et al. 1989 | Not longitudinal studies |
| Haines et al. 1982 | Not reporting wine consumption |
| Seitz et al. 1988 | Review articles, ecological studies, editorials, or case reports |
| Kalapothaki et al. 1993 | Not longitudinal studies |
| Mack et al. 1986 | Not longitudinal studies |
| MacMahon et al. 1982 | Review articles, ecological studies, editorials, or case reports |
| Nkondjock et al. 2005 | Not longitudinal studies |
| Silverman et al. 2001 | Not reporting wine consumption |
| Silverman et al. 1995 | Review articles, ecological studies, editorials, or case reports |
| Soler et al. 1998 | Not longitudinal studies |
| Zatonski et al. 1993 | Not reporting wine consumption |
| Falk et al. 1988 | Not reporting wine consumption |
| Cuzick et al. 1989 | Review articles, ecological studies, editorials, or case reports |
| Ghadirian et al. 1991 | Review articles, ecological studies, editorials, or case reports |
| Pfeffer et al. 1989 | Review articles, ecological studies, editorials, or case reports |
| Friedman et al. 1993 | Not longitudinal studies |
| Harnack et al. 1998 | Not longitudinal studies |
| Heuch et al. 1984 | Not longitudinal studies |
| Hiatt et al. 1983 | Not longitudinal studies |
| Hirayama et al. 1989 | Not reporting wine consumption |
| Ye et al. 2002 | Not longitudinal studies |
| Zheng et al. 1993 | Review articles, ecological studies, editorials, or case reports |
| Hamilton et al. 2000 | Review articles, ecological studies, editorials, or case reports |
| Mason et al. 2005 | Review articles, ecological studies, editorials, or case reports |
| Giovannucci et al. 2004 | Review articles, ecological studies, editorials, or case reports |
| Pietinen et al. 1988 | Review articles, ecological studies, editorials, or case reports |
| Partanen et al. 1997 | Review articles, ecological studies, editorials, or case reports |
| Olsen et al. 1989 | Review articles, ecological studies, editorials, or case reports |
| Ji et al. 1995 | Not reporting wine consumption |
| Rohan et al. 2000 | Not longitudinal studies |
| Sellers et al. 2001 | Review articles, ecological studies, editorials, or case reports |
| Liu et al. 2000 | Review articles, ecological studies, editorials, or case reports |
| Li et al. 2004 | Review articles, ecological studies, editorials, or case reports |
| Vrieling et al. 2009 | Not longitudinal studies |
| Uomo et al. 2007 | Not longitudinal studies |
| Li et al. 2008 | Not longitudinal studies |
| Irving et al. 2009 | Review articles, ecological studies, editorials, or case reports |
| Goswami et al. 2009 | Not longitudinal studies |
| Kueck et al. 2007 | Not longitudinal studies |
| Celik et al. 2007 | Not longitudinal studies |
| Larsson et al. 2004 | Not longitudinal studies |
| Gallus et al. 2007 | Review articles, ecological studies, editorials, or case reports |
| Isaksson et al. 2002 | Review articles, ecological studies, editorials, or case reports |
| Coughlin et al. 2000 | Not longitudinal studies |
| Lieber et al. 1994 | Not longitudinal studies |
| Tworoger et al. 2008 | Not longitudinal studies |
| Singletary et al. 2001 | Review articles, ecological studies, editorials, or case reports |
| Rimando et al. 2002 | Review articles, ecological studies, editorials, or case reports |
| Wolter et al. 2002 | Not longitudinal studies |
| Pignatelli et al. 2006 | Review articles, ecological studies, editorials, or case reports |
| Stockley et al. 2012 | Review articles, ecological studies, editorials, or case reports |
| Ferro-Luzzi et al. 1995 | Review articles, ecological studies, editorials, or case reports |
| Smoliga et al. 2011 | Review articles, ecological studies, editorials, or case reports |
| Dennis et al. 2000 | Review articles, ecological studies, editorials, or case reports |
| Sigvardsson et al. 1996 | Not longitudinal studies |
| Dreyer et al. 1997 | Not longitudinal studies |
| Moller et al. 1997 | Not longitudinal studies |
| Morton et al. 1996 | Not longitudinal studies |
| Cerhan et al. 1997 | Not longitudinal studies |
| Hsing et al. 1990 | Not longitudinal studies |
| Pawlega et al. 1996 | Not longitudinal studies |
| Slattery et al. 1993 | Not longitudinal studies |
| Nakata et al. 1993 | Not longitudinal studies |
| West et al. 1991 | Not longitudinal studies |
| Fincham et al. 1990 | Not longitudinal studies |
| Chaklin et al. 1984 | Not longitudinal studies |
| Mishina et al. 1981 | Not longitudinal studies |
| De Stefani et al. 1995 | Not longitudinal studies |
| Talamini et al. 1986 | Not longitudinal studies |
| Niijima et al. 1980 | Not longitudinal studies |
| Wynder et al. 1971 | Not longitudinal studies |
| Zeegers et al. 1999 | Not longitudinal studies |
| Negri et al. 1992 | Not longitudinal studies |
| Negri et al. 1993 | Not longitudinal studies |
| Fioretti et al. 1999 | Not longitudinal studies |
| Dorgan et al. 2001 | Not longitudinal studies |
| Doll et al. 1999 | Not longitudinal studies |
| Bosetti et al. 2000 | Not longitudinal studies |
| Tonnesen et al. 1994 | Not longitudinal studies |
| McCann et al. 2003 | Not longitudinal studies |
| Heuch et al. 1983 | Not longitudinal studies |
| Harnack et al. 1997 | Not reporting wine consumption |
| Nilsen et al. 2000 | Not reporting wine consumption |
| Villeneuve et al. 2000 | Not reporting wine consumption |
| Stolzenberg et al. 2001 | Not reporting wine consumption |
| Lowenfels et al. 1999 | Not longitudinal studies |
| Kelemen et al. 2004 | Not longitudinal studies |
| Lin et al. 2004 | Not longitudinal studies |
| Percy et al. 1990 | Review articles, ecological studies, editorials, or case reports |
| Poschl et al. 2004 | Review articles, ecological studies, editorials, or case reports |
| Scully et al. 1997 | Review articles, ecological studies, editorials, or case reports |
| Kuper et al. 2004 | Not reporting wine consumption |
| Gavaler et al. 1992 | Review articles, ecological studies, editorials, or case reports |
| Bertone et al. 2002 | Not reporting wine consumption |
| Go et al. 2005 | Not longitudinal studies |
| Rimm et al. 1991 | Not reporting wine consumption |
| Gohagan et al. 2000 | Review articles, ecological studies, editorials, or case reports |
| Kato et al. 1997 | Not reporting wine consumption |
| Calle et al. 2002 | Not longitudinal studies |
| Genkinger et al. 2009 | Not longitudinal studies |
| Ye et al. 2002 | Not longitudinal studies |
| Tonnesen et al. 1994 | Not longitudinal studies |
| Sigvardsson et al. 1996 | Not longitudinal studies |
| Jiao et al. 2009 | Not longitudinal studies |
| Suzuki et al. 2008 | Not longitudinal studies |
| Partanen et al. 1997 | Not reporting wine consumption |
| Silverman et al. 1995 | Not longitudinal studies |
| Isaksson et al. 2002 | Not reporting wine consumption |
| Lin et al. 2002 | Not longitudinal studies |
| Michaud et al. 2004 | Review articles, ecological studies, editorials, or case reports |
| Seitz et al. 1985 | Review articles, ecological studies, editorials, or case reports |
| Ferlay et al. 1999 | Review articles, ecological studies, editorials, or case reports |
| Thurston et al. 2005 | Not reporting wine consumption |
| Klipstein et al. 2002 | Not longitudinal studies |
| Sieri et al. 2002 | Not longitudinal studies |
| Slimani et al. 2000 | Not longitudinal studies |
| Kaaks et al. 1997 | Not longitudinal studies |
| Riboli et al. 2002 | Not reporting wine consumption |
| Kristiansen et al. 2008 | Not reporting wine consumption |
| Meyer et al. 1993 | Review articles, ecological studies, editorials, or case reports |
| Boffeta et al. 2006 | Review articles, ecological studies, editorials, or case reports |
| Corrao et al. 2004 | Review articles, ecological studies, editorials, or case reports |
| Moskal et al. 2007 | Review articles, ecological studies, editorials, or case reports |
| Wei et al. 2004 | Review articles, ecological studies, editorials, or case reports |
| Murata et al. 1996 | Not reporting wine consumption |
| Chyou et al. 1996 | Not reporting wine consumption |
| Otani et al. 2003 | Not reporting wine consumption |
| Singh et al. 1998 | Review articles, ecological studies, editorials, or case reports |
| Su et al. 2004 | Not reporting wine consumption |
| Shimizu et al. 2003 | Not reporting wine consumption |

| **Reference** | **Adjusted for** |
| --- | --- |
| **BREAST CANCER** | |
| Harvey et al. 1986 | Adjusted for all other alcohol consumption. |
| Howe et al. 1991 | Adjusted for the classic non-dietary risk factors for breast cancer. |
| Sneyd et al. 1991 | Adjusted for age, parity, social class, smoking and age at menarche. |
| Friedenreich et al.  1993 | Adjusted for age, menopausal status (pre-, postmenopausal), ever smoker (yes/no), family history of breast cancer (yes/no), parity, total caloric intake (all calories besides those from alcohol), and Interaction between ever smoker and menopausal status |
| Longnecker et al. 1995 | Adjusted for age, state, age a first full-term pregnancy, parity, body mass index, age at menarche, education, benign breast disease, and family history of breast cancer. |
| Swanson et al. 1996 | Adjusted for age, study site, race, parity, and oral contraceptive use. |
| Zhang et al. 1999 | Adjusted for education, height, body mass index, physical activity index, age at first pregnancy (Original Cohort only), parity, age at menarche (Offspring Cohort only), age at menopause, average number of cigarettes smoked, post-menopausal estrogen use, and intake of other alcoholic beverage. |
| Rohan et al. 2000 | Adjusted for age, age at menarche, number of live births, menopausal status, family history of breast cancer in a ®rst-degree relative, practice  of breast self-examination, study allocation, study center, and energy intake. |
| Horn-Ross et al. 2002 | Adjusted for age, race, daily caloric intake, family history of breast cancer, age at menarche, nulliparity/age at first full-term pregnancy,  physical activity, and an interaction term for body mass index and menopausal status. |
| Tjønneland et al. 2007 | Adjusted for height (linear), weight (linear), age at menarche, parity (yes/no), current oral contraceptive use (yes/no), current use of hormone  replacement therapy (yes/no), menopausal status (pre-, peri-, postmenopausal), smoking status (current, former, never), and education (none, primary school, technical/ professional school, secondary school, university) |
| Li et al. 2009 | Adjusted for age, ethnicity, education, body mass index, marital status, smoking alcohol, history of any breast surgery, family history and parity; numbers do not add up to 2829 total breast cancer because of the missing values in models BMI = body mass index. |
| **PROSTATE CANCER** | |
| Tavani et al. 1994 | Adjusted for age, center, education, marital status, body mass index, and smoking status. |
| De Stefani et al. 1995 | Adjusted for age, residence, education, cigarette smoking, and dietary items (meat, milk and fruits). |
| Hayes et al. 1996 | Adjusted for age, ethnicity, and study site, |
| Andersson et al. 1996 | Adjusted for age. |
| Jain et al. 1998 | Adjusted for age. |
| Schuurman et al. 1999 | Adjusted for age, socioeconomic status and family history of prostate cancer. |
| Breslow et al. 1999 | Adjusted for birth-cohort effects. |
| Putnam et al. 2000 | Adjusted for age, family history of prostate cancer, body mass index, total energy, and intake of carbohydrate, linoleic acid, lycopene, retinol, and red meat. |
| Barba et al. 2004 | Adjusted for age, cigarette smoking status, education, body mass index (BMI), and waist o hip ratio. |
| Crispo et al. 2004 | Adjusted for total drinks/week |
| Chang et al. 2005 | Adjusted for age (5-year categories), smoking history (ever, never), current body mass index, family history of prostate cancer, and intake of other alcohol types, dairy products, red meat, and fruits and vegetables. |
| Schoonen et al. 2005 | Adjusted for age, use of PSA screening, total lifetime number of female sexual partners and smoking status. |
| Sutcliffe et al. 2007 | Adjusted for age, race/ethnicity, body mass index at age 21, cumulative family history of prostate cancer through 1996, height, updated cigarette smoking in the past 10 yr, baseline intakes of total energy, tomato sauce, red meat, fish, calcium and vitamin E, baseline energy-adjusted intakes of fructose and a-linolenic acid, baseline vigorous physical activity and updated diabetes mellitus type 2 and vasectomy status adjusted for all other specific alcoholic beverage types. |
| **RENAL CELL CANCER** | |
| Pelucchi et al. 2002 | Adjusted for age, study centre, education, body mass index, cigarette smoking, history of bladder infection and consumption of meat, vegetables and fruit. |
| Rashidkhani et al. 2005 | Adjusted for age and body mass index. |
| Greving et al. 2007 | Adjusted for age, sex, BMI (quartiles), cigarette smoking (three categories), and the other six beverages (continuous). |
| Lew et al. 2011 | Adjusted for age, race, body mass index, marital status, education, vigorous physical activity, smoking, history of hypertension, and intakes of protein and total energy excluding energy from alcohol. |
| **PANCREATIC CANCER** | |
| Tavani et al. 1997 | Adjusted for age, sex, education, smoking status, and history of diabetes, pancreatitis, and cholelithiasis |
| Michaud et al. 2001 | Adjusted for age in 5-year categories, pack-years of smoking, BMI, history of diabetes mellitus, history of cholecystectomy, energy intake, and period. |
| Heinen et al. 2009 | Adjusted for age, sex, smoking, energy intake, body mass index, vegetable intake, fruit intake and total ethanol intake. |
| Jiao et al. 2009 | Adjusted for sex, smoking variable, total energy intake, energy-adjusted saturated fat, red meat, and total folate intake, body mass index, physical activity, history of diabetes and use of other types of alcoholic beverages. |
| Gapstur et al. 2011 | Adjusted for age, sex, race/ethnicity, education, marital status, body mass index, family history of pancreatic cancer, and personal history of  gallstones, diabetes mellitus, or smoking. |
| **OVARIAN CANCER** | |
| Gwinn et al. 1986 | Adjusted for age, geographic region, religion, education, smoking, oral contraceptive use, parity, infertility, family history of ovarian cancer, and consumption of other alcoholic beverages. |
| La Vecchia et al. 1992 | Adjusted for age, education, smoking, menstrual and reproductive factors, oral contraceptive use and indicators of fat and green vegetable consumption. |
| Tavani et al. 2001 | Adjusted for study center, year of interview, age, education, parity, age at menopause, oral contraceptive use, family history of ovarian/breast cancer,  body mass index, and energy intake (without alcohol calories). |
| Webb et al. 2003 | Adjusted for age, age squared, level of education, BMI, smoking status, duration of OC use, parity, and caffeine intake, consumption of other types of alcohol. |
| Goodman et al. 2003 | Adjusted by unconditional multiple logistic regression model for age, ethnicity, education, study site, use of oral contraceptives, parity, and tubal ligation. |
| Modugno et al. 2003 | Adjusted by for age, parity, oral contraceptive use, education, tubal ligation, smoking, and family history of ovarian cancer. |
| Schouten et al. 2004 | Adjusted for age, use of oral contraceptives, parity, height, body mass index, total energy intake, and current cigarette smoking. |
| Peterson et al. 2006 | Adjusted for age, state of residence and grams of other types of alcohol consumed. |
| Chang et al. 2007 | Adjusted for race, total energy intake, parity, oral contraceptive use, strenuous exercise, and menopausal status/hormone therapy use, stratified by age at baseline and consumption of other alcohol types in the same time period. |
| Cook et al. 2016 | Adjusted for study site, diagnosis/reference age, OC use, parity, current smoking, and family history of ovarian or breast cancer. |
| **AERODIGESTIVE TRACT CANCERS** | |
| Franceschi et al. 1990 | Adjusted for age, area of residence, years of education, occupation and smoking habit. |
| De Stefani et al. 1998 | Adjusted for age, residence, urban/rural status, education and tobacco smoking. |
| Grønbæk et al. 1998 | Adjusted for age, sex, smoking habits and educational level. |
| Huang et al. 2003 | Adjusted for age, tobacco use, consumption of raw fruits and vegetables, educational level, and total alcohol intake. |
| Barstad et al. 2005 | Adjusted for age, gender and smoking. |
| Pandeya et al. 2009 | Adjusted for age, sex, body mass index 1 year ago, frequency of heartburn or acid reflux, education, frequency of aspirin use in the past 5 years and smoking. |
| **SKIN CARCINOMA** | |
| Fung et al. 2002 | Adjusted for tendency to tan, tendency to sunburn, natural hair color at age 20, major ancestry, and number of lifetime blistering sunburns, the use of sunscreen, state of residence at age 15, a proxy of sun exposure in childhood, current state of residence, a proxy for recent sun exposure (in the NHS). In the HPFS, adjusted for major ancestry, natural hair color at age 18, eye color, tendency to burn or tan in adolescence, state of residence at 15, and current state of residence. In both algo adjusted for age and BMI. |
| Ansems et al. 2008 | Adjusted for age, sex, beta-carotene treatment, sunscreen treatment, self-reported skin colour, number of pack-years smoked until 1992, elastosis of the neck, leisure time sun exposure and history of skin cancer before 1992. |
| **LUNG CANCER** | |
| Benedetti et al. 2006 | Adjusted for age, respondent status, ethnicity, smoking status, cigarette-years, socio-economic status, years of schooling, and time since quitting. |
| **COLORECTAL CANCER** | |
| Potter et al. 1986 | Not adjusted. |
| Longnecker et al. 1990 | Adjusted for age, smoking, and income slightly increased the relative risks. |
| Freudenheim et al. 1990 | Adjusted for kilocalories, fat, dietary fiber, vitamin C or carotenoids. |
| Meyer et al. 1993 | Adjustment for age, interviewer, dietary energy intake, and alcohol consumption. |
| Newcomb et al. 1993 | Adjusted to age distribution of the cases. |
| Gapstur et al. 1994 | Adjusted for age and indicator variables for each type of alcoholic beverage. |
| Goldbohm et al. 1994 | Adjusted for age. |
| Sharpe et al. 2002 | Adjusted for the cumulative consumption of both other types of alcohol, and for age, respondent status, ethnicity, family income, years of education, marital status, and cigarette smoking. |
| Pedersen et al. 2003 | Adjusted for age, sex, smoking, body mass index, study of origin, and alcohol intake of the remaining two types of alcohol. |
| Bongaerts et al. 2008 | Adjusted for age, sex, family history of CRC, body mass index, nonoccupational physical activity, total energy intake, and energy-adjusted intakes of fat (g/day), dietary fiber (g/day) and calcium (mg/day), and total alcohol consumption. |
| Crockett et al. 2011 | Adjusted for age, sex, race, red meat intake, NSAID use, family history of colorectal cancer, obesity, smoking status, and education level. |
| **CANCER IN GENERAL** | |
| Gong et al. 2009 | Adjusted for age, race, body mass index, and other types of beverage intake for each specific type of alcohol intake. |
| Smyth et al. 2015 | Adjusted for age, sex, smoking, ethnicity, education, body-mass index, diabetes, hypertension, jaundice or hepatitis, physical activity, diet (dairy, fruits, vegetables, meats, fish, soft drinks, processed foods, nuts, and trans fats), medications (angiotensin-converting enzyme inhibitors or angiotensin receptor blockers, β blockers, calcium channel blockers, diuretics, α blockers, lipid-lowering therapy, and anti-thrombotic therapy), wealth index, total alcohol consumption (drinks per day), and heavy episodic drinking pattern. |
| Schutte et al. 2021 | Adjusted for baseline age, body mass index, sex, smoking, systolic blood pressure, physical activity, diabetes and Townsend deprivation index by quintiles of the distribution of weekly alcohol intake in grams per week with never drinkers or the first quintile as reference. |
| **GLIOMA** | |
| Ryan et al. 1992 | Adjustment for age, sex and the subject's own smoking status. |
| Hurley et al. 1996 | Adjusted for age, a reference date and gender. |
| Efird et al. 2004 | Adjusting for cigarettes, cigars, pipes, sex, race, education, alcohol and coffee. |

**Table S3.** Adjustments to the results of each study.

**Table S4.** Quality assessment using the Newcastle-Ottawa Quality Assessment Scale for Cohort studies.

| Reference | (a) Selection | | | | (b) Comparability | | (c) Outcome | | | Total (a+b+c) |
| --- | --- | --- | --- | --- | --- | --- | --- | --- | --- | --- |
|  | 1 | 2 | 3 | 4 | 1 | 2 | 1 | 2 | 3 |  |
| Gapstur et al. 1994 |  |  |  |  |  |  |  |  |  | 8 |
| Goldbohm et al. 1994 |  |  |  |  |  |  |  |  |  | 7 |
| Grønbæk et al. 1998 |  |  |  |  |  |  |  |  |  | 9 |
| Breslow et al. 1999 |  |  |  |  |  |  |  |  |  | 9 |
| Schuurman et al. 1999 |  |  |  |  |  |  |  |  |  | 9 |
| Zhang et al. 1999 |  |  |  |  |  |  |  |  |  | 9 |
| Rohan et al. 2000 |  |  |  |  |  |  |  |  |  | 9 |
| Putnam et al. 2000 |  |  |  |  |  |  |  |  |  | 8 |
| Michaud et al. 2001 |  |  |  |  |  |  |  |  |  | 7 |
| Fung et al. 2002 |  |  |  |  |  |  |  |  |  | 7 |
| Horn-Ross et al. 2002 |  |  |  |  |  |  |  |  |  | 8 |
| Pedersen et al. 2003 |  |  |  |  |  |  |  |  |  | 8 |
| Efird et al. 2004 |  |  |  |  |  |  |  |  |  | 8 |
| Schouten et al. 2004 |  |  |  |  |  |  |  |  |  | 8 |
| Platz et al. 2004 |  |  |  |  |  |  |  |  |  | 9 |
| Barstad et al. 2005 |  |  |  |  |  |  |  |  |  | 9 |
| Rashidkhani et al. 2005 |  |  |  |  |  |  |  |  |  | 7 |
| Chang et al. 2007 |  |  |  |  |  |  |  |  |  | 7 |
| Sutçcliffe et al. 2007 |  |  |  |  |  |  |  |  |  | 7 |
| Tjønneland et al. 2007 |  |  |  |  |  |  |  |  |  | 8 |
| Ansems et al. 2008 |  |  |  |  |  |  |  |  |  | 8 |
| Bongaerts et al. 2008 |  |  |  |  |  |  |  |  |  | 8 |
| Gong et al. 2009 |  |  |  |  |  |  |  |  |  | 8 |
| Heinen et al. 2009 |  |  |  |  |  |  |  |  |  | 8 |
| Jiao et al. 2009 |  |  |  |  |  |  |  |  |  | 8 |
| Li et al. 2009 |  |  |  |  |  |  |  |  |  | 9 |
| Baglietto et al. 2011 |  |  |  |  |  |  |  |  |  | 9 |
| Gapstur et al. 2011 |  |  |  |  |  |  |  |  |  | 8 |
| Lew et al. 2011 |  |  |  |  |  |  |  |  |  | 8 |
| Smyth et al. 2015 |  |  |  |  |  |  |  |  |  | 8 |
| Schutte et al. 2021 |  |  |  |  |  |  |  |  |  | 7 |

**Table S5.** Quality assessment using the Newcastle-Ottawa Quality Assessment Scale for Case-control studies.

| Reference | (a) Selection | | | | (b) Comparability | | (c) Outcome | | | Total (a+b+c) |
| --- | --- | --- | --- | --- | --- | --- | --- | --- | --- | --- |
|  | 1 | 2 | 3 | 4 | 1 | 2 | 1 | 2 |  |  |
| Gwinn et al. 1986 |  |  |  |  |  |  |  |  |  | 8 |
| Harvey et al. 1986 |  |  |  |  |  |  |  |  |  | 8 |
| Potter et al. 1986 |  |  |  |  |  |  |  |  |  | 8 |
| Barra et al. 1990 |  |  |  |  |  |  |  |  |  | 7 |
| Franceschi et al. 1990 |  |  |  |  |  |  |  |  |  | 6 |
| Freudenheim et al. 1990 |  |  |  |  |  |  |  |  |  | 7 |
| Longnecker et al. 1990 |  |  |  |  |  |  |  |  |  | 8 |
| Barra et al. 1991 |  |  |  |  |  |  |  |  |  | 7 |
| Howe et al. 1991 |  |  |  |  |  |  |  |  |  | 6 |
| Sneyd et al. 1991 |  |  |  |  |  |  |  |  |  | 8 |
| La Vecchia et al. 1992 |  |  |  |  |  |  |  |  |  | 7 |
| Ryan et al. 1992 |  |  |  |  |  |  |  |  |  | 8 |
| Friedenreich et al. 1993 |  |  |  |  |  |  |  |  |  | 8 |
| Meyer et al. 1993 |  |  |  |  |  |  |  |  |  | 8 |
| Newcomb et al. 1993 |  |  |  |  |  |  |  |  |  | 8 |
| Tavani et al. 1994 |  |  |  |  |  |  |  |  |  | 8 |
| Longnecker et al. 1995 |  |  |  |  |  |  |  |  |  | 8 |
| De Stefani et al. 1995 |  |  |  |  |  |  |  |  |  | 8 |
| Hurley et al. 1996 |  |  |  |  |  |  |  |  |  | 8 |
| Swanson et al. 1996 |  |  |  |  |  |  |  |  |  | 8 |
| Hayes et al. 1996 |  |  |  |  |  |  |  |  |  | 8 |
| Andersson et al. 1996 |  |  |  |  |  |  |  |  |  | 8 |
| Tavani et al. 1997 |  |  |  |  |  |  |  |  |  | 7 |
| De Stefani et al. 1998 |  |  |  |  |  |  |  |  |  | 7 |
| Jain et al. 1998 |  |  |  |  |  |  |  |  |  | 8 |
| Tavani et al. 2001 |  |  |  |  |  |  |  |  |  | 8 |
| Pelucchi et al. 2002 |  |  |  |  |  |  |  |  |  | 7 |
| Sharpe et al. 2002 |  |  |  |  |  |  |  |  |  | 6 |
| Goodman et al. 2003 |  |  |  |  |  |  |  |  |  | 8 |
| Huang et al. 2003 |  |  |  |  |  |  |  |  |  | 8 |
| Modugno et al. 2003 |  |  |  |  |  |  |  |  |  | 8 |
| Webb et al. 2003 |  |  |  |  |  |  |  |  |  | 7 |
| Barba et al. 2004 |  |  |  |  |  |  |  |  |  | 8 |
| Chang et al. 2005 |  |  |  |  |  |  |  |  |  | 7 |
| Crispo et al. 2004 |  |  |  |  |  |  |  |  |  | 7 |
| Benedetti et al. 2006 |  |  |  |  |  |  |  |  |  | 8 |
| Schoonen et al. 2005 |  |  |  |  |  |  |  |  |  | 8 |
| Peterson et al. 2006 |  |  |  |  |  |  |  |  |  | 7 |
| Greving et al. 2007 |  |  |  |  |  |  |  |  |  | 7 |
| Pandeya et al. 2009 |  |  |  |  |  |  |  |  |  | 7 |
| Crockett et al. 2011 |  |  |  |  |  |  |  |  |  | 8 |
| Cook et al. 2016 |  |  |  |  |  |  |  |  |  | 6 |

**Table S6.** Sensitivity analysis.

| **BREAST CANCER** | | | |
| --- | --- | --- | --- |
| **Reference** | **RR** | **LL** | **UL** |
| Friedenreich et al. 1993 (Premenopausal) | 0,018 | -0,001 | 0,037 |
| Friedenreich et al. 1993 (Postmenopausal) | 0,019 | 0,000 | 0,038 |
| Zhang et al. 1999 (Framingham study) | 0,019 | -0,000 | 0,037 |
| Zhang et al. 1999 (Framingham Offspring Study) | 0,019 | 0,000 | 0,037 |
| Rohan et al. 2000 | 0,018 | -0,000 | 0,037 |
| Tjønneland et al. 2007 | 0,017 | -0,007 | 0,041 |
| Li et al. 2009 | 0,016 | -0,013 | 0,044 |

RR: risk ratio; LL: low limit; UL: upper limit.

| **OVARIAN CANCER** | | | |
| --- | --- | --- | --- |
| **Reference** | **RR** | **LL** | **UL** |
| La Vecchia et al. 1992 | -0.033 | -0.162 | 0.097 |
| Tavani et al. 2001 | -0.029 | -0.159 | 0.101 |
| Goodman et al. 2003 (Mucinous) | 0.004 | -0.111 | 0.119 |
| Goodman et al. 2003 (Invasive ovarian cancer mucinous) | 0.010 | -0.104 | 0.124 |
| Modugno et al. 2003 (Mucinous) | 0.049 | -0.049 | 0.148 |
| Goodman et al. 2003 (Nonmucinous) | 0.015 | -0.101 | 0.130 |
| Modugno et al. 2003 (Nonmucinous) | -0.013 | -0.141 | 0.115 |
| Goodman et al. 2003 (Invasive ovarian cancer serous) | 0.006 | -0.111 | 0.122 |
| Goodman et al. 2003 (Invasive ovarian cancer endometrioid) | 0.028 | -0.081 | 0.137 |
| Schouten et al. 2004 | -0.003 | -0.125 | 0.119 |
| Chang et al. 2007 | -0.027 | -0.142 | 0.088 |
| Cook et al. 2016 (Red wine) | 0.033 | -0.069 | 0.135 |
| Cook et al. 2016 (White wine) | 0.015 | -0.098 | 0.127 |

RR: risk ratio; LL: low limit; UL: upper limit.

| **RENAL CANCER** | | | |
| --- | --- | --- | --- |
| **Reference** | **RR** | **LL** | **UL** |
| Tavani et al. 1994 | -0,098 | -0,230 | 0,033 |
| Schuurman et al. 1999 | -0,109 | -0,238 | 0,020 |
| Crispo et al. 2004 | -0,084 | -0,229 | 0,062 |
| Crispo et al. 2004 | -0,036 | -0,131 | 0,058 |
| Pelucchi et al. 2002 (Women) | -0,088 | -0,223 | 0,046 |
| Pelucchi et al. 2002 (Men) | -0,077 | -0,217 | 0,063 |
| De Stefani et al. 1995 | -0,114 | -0,238 | 0,010 |
| Breslow et al. 1999 | -0,078 | -0,213 | 0,057 |
| Lew et al. 2011 | -0,094 | -0,238 | 0,051 |

RR: risk ratio; LL: low limit; UL: upper limit.

| **COLORECTAL CANCER** | | | |
| --- | --- | --- | --- |
| **Reference** | **RR** | **LL** | **UL** |
| Freudenheim et al. 1990 (Men) | -0,071 | -0,187 | 0,046 |
| Freudenheim et al. 1990 (Women) | -0,087 | -0,203 | 0,029 |
| Gapstur et al. 1994 (Rectal cancer) | -0,075 | -0,189 | 0,039 |
| Sharpe et al. 2002 (Rectal cancer) | -0,110 | -0,216 | -0,004 |
| Pedersen et al. 2003 (Rectal cancer) | -0,084 | -0,203 | 0,036 |
| Bongaerts et al. 2008 (Rectal cancer) | -0,069 | -0,186 | 0,047 |
| Crockett et al. 2011 (Rectal cancer) | -0,064 | -0,178 | 0,049 |
| Bongaerts et al. 2008 (Rectosigmoid cancer) | -0,073 | -0,191 | 0,044 |
| Crockett et al. 2011 (Rectosigmoid cancer) | -0,067 | -0,180 | 0,046 |
| Crockett et al. 2011 (Sigmoid cancer) | -0,064 | -0,178 | 0,050 |
| Pedersen et al. 2003 (Colon cancer) | -0,081 | -0,205 | 0,042 |
| Bongaerts et al. 2008 (Colon cancer) | -0,088 | -0,214 | 0,039 |
| Gapstur et al. 1994 (Proximal colon) | -0,079 | -0,194 | 0,035 |
| Sharpe et al. 2002 (Proximal colon) | -0,085 | -0,201 | 0,030 |
| Bongaerts et al. 2008 (Proximal colon) | -0,078 | -0,201 | 0,045 |
| Gapstur et al. 1994 (Distal colon) | -0,066 | -0,179 | 0,046 |
| Sharpe et al. 2002 (Distal colon) | -0,119 | -0,208 | -0,029 |
| Bongaerts et al. 2008 (Distal colon) | -0,093 | -0,213 | 0,027 |

RR: risk ratio; LL: low limit; UL: upper limit.

**Table S7.** Subgroup analysis according to continent.

| Type of cancer | Continente | N | RR | LL | UL | I^2^ | p |
| --- | --- | --- | --- | --- | --- | --- | --- |
| Breast | Europe | 1 | 1.02 | 0.99 | 1.05 | - | - |
|  | America | 6 | 1.02 | 0.99 | 1.04 | 0.0 | 0.597 |
|  | Overall | 7 | 1.02 | 1.00 | 1.04 | 0.0 | 0.718 |
| Ovarian | Europe | 3 | 1.14 | 1.04 | 1.24 | 0.0 | 0.593 |
|  | America | 10 | 0.90 | 0.75 | 1.08 | 60.8 | 0.006 |
|  | Overall | 13 | 1.01 | 0.90 | 1.12 | 53.9 | 0.011 |
| Renal | Europe | 6 | 0.89 | 0.76 | 1.04 | 54.4 | 0.052 |
|  | America | 3 | 0.97 | 0.85 | 1.11 | 0.0 | 0.429 |
|  | Overall | 9 | 0.92 | 0.81 | 1.04 | 52.8 | 0.030 |
| Colorectal | Europe | 8 | 0.99 | 0.86 | 1.13 | 77.4 | 0.000 |
|  | America | 10 | 0.84 | 0.69 | 1.02 | 62.3 | 0.005 |
|  | Overall | 18 | 0.92 | 0.82 | 1.03 | 71.4 | 0.000 |

RR: risk ratio; LL: low limit; UL: upper limit. N: number of studies.

**Table S8.** Random-effects meta-regression models.

|  | Coefficient | 95%ICs | P value |
| --- | --- | --- | --- |
| Breast cancer | | | |
| Mean age | -0.005 | -0.085, 0.076 | 0.598 |
| Percentage of female | NA | NA | NA |
| Follow-up | - | - | - |
| Ovarian cancer | | | |
| Mean age | -0.043 | -0.112, 0.026 | 0.190 |
| Percentage of female | NA | NA | NA |
| Follow-up | -0.035 | -0.133, 0.062 | 0.443 |
| Renal cancer | | | |
| Mean age | -0.008 | -0.115, 0.098 | 0.818 |
| Percentage of female | NA | NA | NA |
| Follow-up | -0.072 | -0.142, -0.001 | **0.048** |
| Colorectal cancer |  |  |  |
| Mean age | - | - | - |
| Percentage of female | -0.000 | -0.004, 0.004 | 0.985 |
| Follow-up | 0.025 | 0.004, 0.046 | **0.023** |

NA: Non applicabile; IC: Confidence intervals.
